# Proteomic Analysis of Urinary Extracellular Vesicles Reveals a Role for the Complement System in Medullary Sponge Kidney Disease

**DOI:** 10.3390/ijms20215517

**Published:** 2019-11-05

**Authors:** Maurizio Bruschi, Simona Granata, Giovanni Candiano, Antonia Fabris, Andrea Petretto, Gian Marco Ghiggeri, Giovanni Gambaro, Gianluigi Zaza

**Affiliations:** 1 Laboratory of Molecular Nephrology, IRCCS Istituto Giannina Gaslini, 16147 Genova, Italy; mauriziobruschi@gaslini.org (M.B.); giovannicandiano@gaslini.org (G.C.); 2 Renal Unit, Department of Medicine, University/Hospital of Verona, Piazzale A. Stefani 1, 37126 Verona, Italy; simona.granata@univr.it (S.G.); antoniafabris21@gmail.com (A.F.); giovanni.gambaro@univr.it (G.G.); 3 Laboratory of Mass Spectrometry—Core Facilities, IRCCS Istituto Giannina Gaslini, 16147 Genova, Italy; 4 Division of Nephrology, Dialysis and Transplantation, IRCCS Istituto Giannina Gaslini, 16147 Genova, Italy; GMarcoGhiggeri@gaslini.org

**Keywords:** medullary sponge kidney, idiopathic calcium nephrolithiasis, complement system, proteomics

## Abstract

Medullary sponge kidney (MSK) disease is a rare and neglected kidney condition often associated with nephrocalcinosis/nephrolithiasis and cystic anomalies in the precalyceal ducts. Little is known about the pathogenesis of this disease, so we addressed the knowledge gap using a proteomics approach. The protein content of microvesicles/exosomes isolated from urine of 15 MSK and 15 idiopathic calcium nephrolithiasis (ICN) patients was investigated by mass spectrometry, followed by weighted gene co-expression network analysis, support vector machine (SVM) learning, and partial least squares discriminant analysis (PLS-DA) to select the most discriminative proteins. Proteomic data were verified by ELISA. We identified 2998 proteins in total, 1764 (58.9%) of which were present in both vesicle types in both diseases. Among the MSK samples, only 65 (2.2%) and 137 (4.6%) proteins were exclusively found in the microvesicles and exosomes, respectively. Similarly, among the ICN samples, only 75 (2.5%) and 94 (3.1%) proteins were exclusively found in the microvesicles and exosomes, respectively. SVM learning and PLS-DA revealed a core panel of 20 proteins that distinguished extracellular vesicles representing each clinical condition with an accuracy of 100%. Among them, three exosome proteins involved in the lectin complement pathway maximized the discrimination between MSK and ICN: Ficolin 1, Mannan-binding lectin serine protease 2, and Complement component 4-binding protein β. ELISA confirmed the proteomic results. Our data show that the complement pathway is involved in the MSK, revealing a new range of potential therapeutic targets and early diagnostic biomarkers.

## 1. Introduction

In the last decade, great efforts have been undertaken to characterize the biological networking associated with medullary sponge kidney (MSK) disease, a rare clinical condition (prevalence of approximately 5 cases per 100,000 in the general population) often associated with nephrocalcinosis and nephrolithiasis, urinary acidification and concentration defects, and cystic anomalies in the precalyceal ducts [1]. Most of these studies, based on clinical observations, as well as molecular analysis, have provided evidence that supports the genetic transmission of MSK and an association with metabolic disorders (such as hyperparathyroidism) and bone diseases [2,3,4,5,6,7,8,9,10,11].

More recently, we used proteomic analysis to catalog the MSK-specific protein profile, as well as the protein content of urinary extracellular vesicles [12]. This revealed the presence of several key regulators of epithelial cell differentiation, kidney development, cell migration/adhesion, and extracellular matrix organization, providing a new insight into the pathophysiology of MSK [12]. The most abundant protein found in MSK urinary extracellular vesicles was laminin subunit α2 (LAMA2, Merosin), a well-characterized member of the laminin family of at least 15 αβɣ heterotrimeric proteins, which contributes to the extracellular matrix and is a major component of the basement membrane [12,13]. This protein is thought to promote cyst formation [14,15,16]. It also regulates extracellular laminin assembly, and the laminin then determines the orientation of the apical pole [17].

In our most recent publication [18], we compared the protein content of extracellular vesicles (exosomes and microvesicles) from MSK and autosomal dominant polycystic kidney disease (ADPKD) patients by mass spectrometry (MS) and identified a profile including 34 proteins that discriminate between the two clinical conditions. The most abundant protein in MSK vesicles was SPP1, also known as osteopontin, which is implicated in physiological/pathological bone mineralization and kidney stone formation [19]. This protein is synthesized in the kidney and secreted into the urine by epithelial cells, including those lining the loop of Henle, distal convoluted tubule, and papillary duct [20]. SPP1 inhibits the nucleation, growth, and aggregation of calcium oxalate crystals [21] and the binding of these crystals to kidney epithelial cells [22]. Although it has been recently found that osteopontin was up-regulated in the kidney from established rat models for polycystic kidney disease compared with wild-type mice [23,24], no data are available for MSK (no animal model is available for this disease). Therefore, based on our in vivo data, we can assume that in MSK patients, osteopontin could be also more up-regulated than ADPKD. A reason could be that in MSK, this biological element may be implicated not only in cystic development, but also in nephrolithiasis, a major clinical condition associated with medullary sponge kidney.

The diagnostic accuracy for MSK is currently low because it depends on personal experience (MSK is often confused with other causes of nephrocalcinosis or with papillary ductal plugging) and diagnosis requires invasive radiation and/or nephrotoxic contrast agents for medical imaging. Although the studies described above have shed light on the biological mechanisms associated with MSK, the pathogenesis of this disorder is only partially defined and further studies are necessary to identify suitable diagnostic biomarkers. To address this knowledge gap, we carried out a comprehensive comparative proteomic analysis of urinary microvesicles and exosomes to identify biological differences between MSK and idiopathic calcium nephrolithiasis (ICN) as a control group. The analysis of urinary extracellular vesicles is ideal because they can be obtained without invasive procedures and they contain elevated levels of cell-specific proteins from every segment of the nephron, representing diverse cellular processes including metabolic, immunity-related, and coagulation responses [25,26]. These intrinsic characteristics of extracellular vesicles could, therefore, provide a panel of informative marker proteins that not only allow the diagnosis and monitoring of MSK but could also provide insight into the underlying pathophysiological and biochemical processes.

## 2. Results

### 2.1. Characterization of Exosomes and Microvesicles

The size and purity of the microvesicles and exosomes isolated by ultracentrifugation were confirmed by dynamic light scattering (DLS), revealing a Gaussian distribution profile with peak means at 1000 ± 65 and 90 ± 5 nm, the typical size for microvesicles and exosomes, respectively (Figure S1A). There was no difference in size between the MSK and ICN patients for either type of vesicle. Western blot analysis revealed that the exosomes were positive for CD63 and CD81 but not CD45, whereas the microvesicles showed the opposite antigen profile (Figure S1B).

### 2.2. Protein Composition of Exosomes and Microvesicles

**Figure 1:**
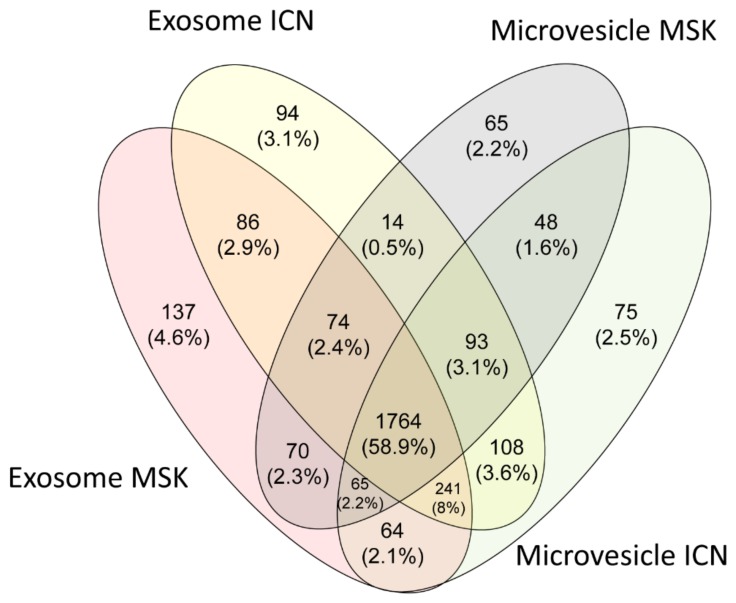
Venn diagram showing all the proteins identified in exosomes and microvesicles isolated from the urine of idiopathic calcium nephrolithiasis (ICN) and medullary sponge kidney (MSK) patients. Venn diagram shows common and exclusive proteins in the ICN and MSK groups. The numbers (and percentages) of proteins in the overlapping and non-overlapping areas are indicated.

The protein composition of microvesicles and exosomes from the urine of ICN and MSK patients was determined by MS analysis. We identified 2998 proteins in total, 1764 (58.9%) of which were present in all four sample types. Among the ICN samples, only 75 (2.5%) and 94 (3.1%) proteins were exclusively found in the microvesicles and exosomes, respectively. Similarly, among the MSK samples, only 65 (2.2%) and 137 (4.6%) proteins were exclusively found in the microvesicles and exosomes, respectively (Figure 1).

About 2% of the total proteins found in extracellular vesicles were associated with one or both kidney diseases according to the DisGeNET database [27]. Among these associated proteins, 40% were found in the ICN and 100% in the MSK samples (Figure S2). The cellular origins of the proteins in the microvesicles were very similar in the ICN and MSK disease samples, with 35% of proteins originating from membranes, 25% from the cytoplasm, 7% from the nucleus, and 33% from other organelles. Similar results were observed for the exosome proteins, with 19% originating from membranes, 31% from the cytoplasm, 11% from the nucleus, and 38% from other organelles.

**Figure 2:**
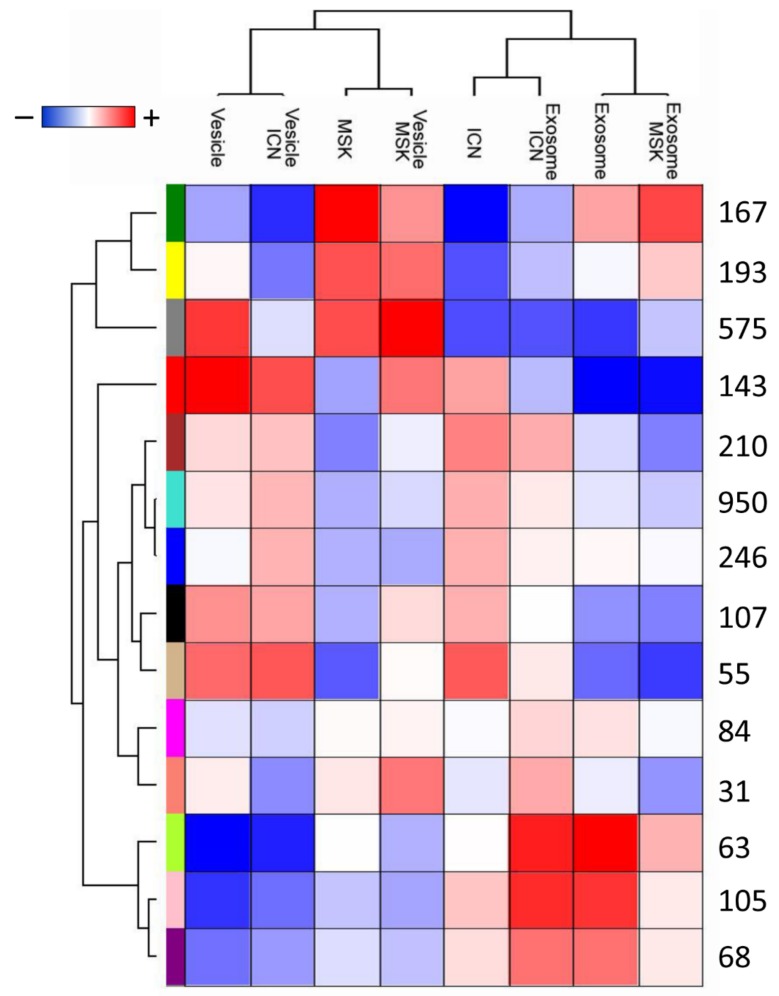
Module identification and relationships with clinical traits. Heat map of the relationships between module eigengenes and the trait indicator of each sample. The grade of the relationship ranges from −1 (blue) to 1 (red), where blue represents a perfect negative correlation and red a perfect positive correlation.

The overlapping protein content of each sample was confirmed by constructing a two-dimensional scatter plot of the multidimensional scaling (MDS) analysis (Figure S3). No samples were excluded during the quality check, performed by non-hierarchical clustering. We used weighted-gene co-expression network analysis (WGCNA) to identify proteins associated with each type of extracellular vesicle and disease, revealing a total of 14 modules comprising proteins with similar expression profiles. To distinguish between modules, we chose an arbitrary color for each module. The number of proteins included in each module ranged from 31 (salmon) to 950 (turquoise). The lime, pink, violet, and tan modules showed closer relationships with the microvesicles or exosomes from the ICN samples, whereas the gray, yellow, and green modules showed closer relationships with the microvesicles from the MSK samples (Figure 2).

**Figure 3:**
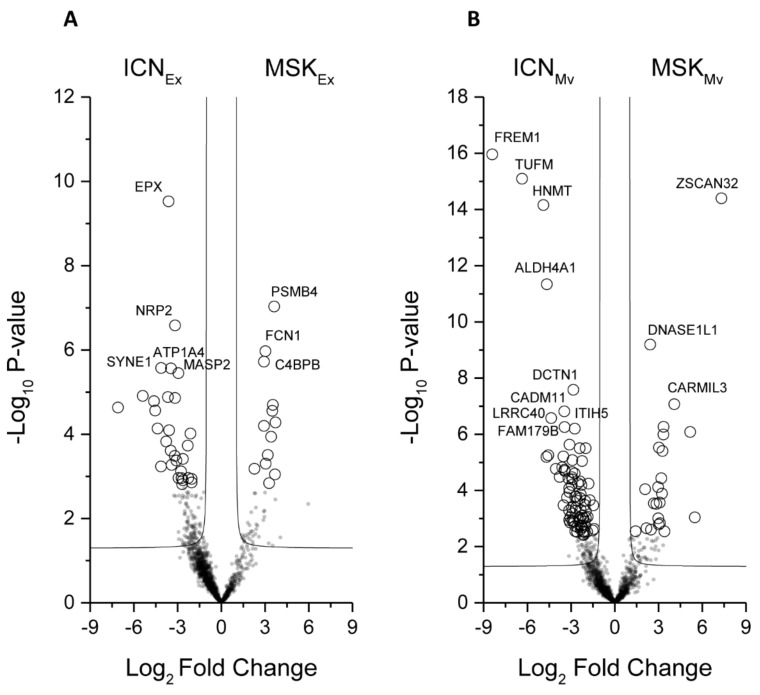
Volcano plot showing the univariate statistical analysis of urinary exosome (**A**) and microvesicle (**B**) fractions from ICN and MSK patients. The plot is based on the fold change (log_2_) and p-value (–log_10_) of all proteins identified in all samples. White circles indicate proteins with statistically significant differences in abundance between the two groups of patients.

Next, we applied the Mann–Whitney *U*-test to identify the proteins that best distinguish the type of disease in the microvesicles or exosomes (Figure 3A,B).

**Figure 4:**
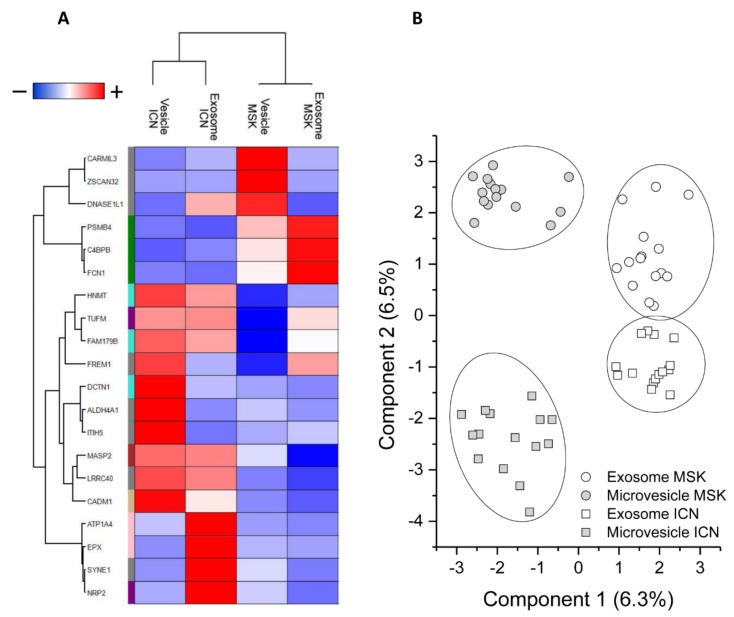
Proteins that achieve maximum discrimination between the exosome and microvesicle fractions of ICN and MSK patients. (**A**) Heat map of 20 core proteins identified through the combined use of univariate statistical analysis, machine learning, and partial least squares discriminant analysis. In the heat map, each row represents a protein, and each column corresponds to a sample type (exosome and microvesicle fractions from ICN and MSK patients). Normalized Z-scores of protein abundance are depicted by a pseudocolor scale with red indicating positive expression, white equal expression, and blue negative expression compared to each protein value, whereas the dendrogram displays the outcome of unsupervised hierarchical clustering, placing similar proteome profile values near each other. (**B**) Two-dimensional scatter plot representing the partial least squares discriminant analysis of exosomes (white symbols) and microvesicles (gray symbols) from MSK (circle) and ICN (square) patients, using the 20 core proteins. The ellipsis indicates 95% confidence interval. Visual inspection of the dendrogram, heat map, and scatter plot confirm the ability of these proteins to clearly distinguish among the four different sample types.

This revealed a total of 142 discriminatory proteins (Tables S1 and S2): 105 distinguished between ICN and MSK microvesicles and 43 distinguished between ICN and MSK exosomes, with six featuring in both vesicle types (Figure S4A). Their expression profiles after Z-score analysis are shown in Figure S4B. support vector machine (SVM) learning and partial least squares discriminant analysis (PLS-DA) were then used to highlight the proteins that maximize the discrimination between different sample types, revealing a core panel of 20 proteins that identified the four sample types with an accuracy of 100% (Figure 4A). Following the Z-score analysis, we built a heat map of the corresponding expression profiles (Figure 4B).

**Figure 5:**
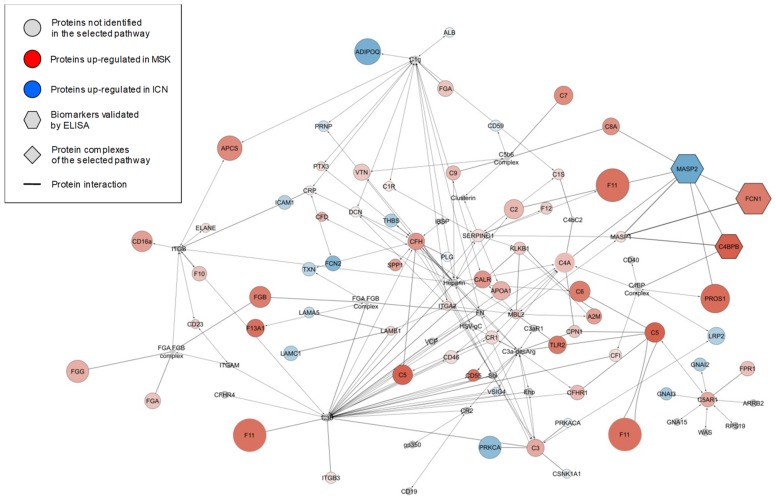
Complement lectin pathway activation. The diagram shows the zoom-in of the immune system enrichment results mapped as a network. Nodes and edges represent, respectively, the proteins and their interaction in the pathway of complement lectin activation. The color intensity of each protein (node) indicates the fold change increment in the urinary exosome fraction of MSK (red) versus ICN (blue) patients and the node size is representative of their p-value. The gray nodes represent unidentified proteins (circle) and protein complexes (diamonds) in the lectin pathway. The hexagons indicate the three proteins that maximize the discrimination between ICN and MSK samples as highlighted by the combined use of univariate/multivariate statistical analysis and machine learning algorithms.

The diversity of expression profiles among the proteins that showed significant and high levels of sample discrimination indicated an equally diverse range of functions, so we assigned Gene Ontology (GO) functional annotations to build a network of biochemical pathways among the different groups (Figure S5). In this network, circles and lines represent the biochemical pathways and their interconnections, respectively. In addition, the different pathways were clustered into 12 functional groups based on their GO annotations (ellipses): immune system, production of reactive oxygen species, IL-8 regulation, transport, regulation of ERBB signaling, tyrosine phosphorylation, receptor activity, peptidyl serine modification, hydrolase activity, DNA regulation, nucleoside metabolism, and cell and organ development. These 12 clusters were grouped into four macro-areas: regulation of metabolism, signal transduction, inflammation, and regulation of cell development. To generate a more concise picture of the biochemical process associated with the three proteins that maximize the discrimination between the exosomes of ICN and MSK samples [Mannan-binding lectin serine protease 2 (MASP2), Ficolin 1 (FCN1), and Complement component 4-binding protein β (C4BPB)], we explored the enrichment of GO annotations in greater detail. This highlighted the lectin-based complement activation pathway as the perturbed biochemical process most likely associated with all three potential biomarkers (Figure 5). The diagram shows the pathway mapped as a network of proteins (nodes) and their interactions (edges). Node color represents the fold change in protein abundance in the urinary exosome fraction of MSK (red) versus ICN (blue) patients, and the node size represents the corresponding p-values.

### 2.3. ELISA Analysis of MASP2, FCN1, and C4BPB to Confirm the MS Data

**Figure 6:**
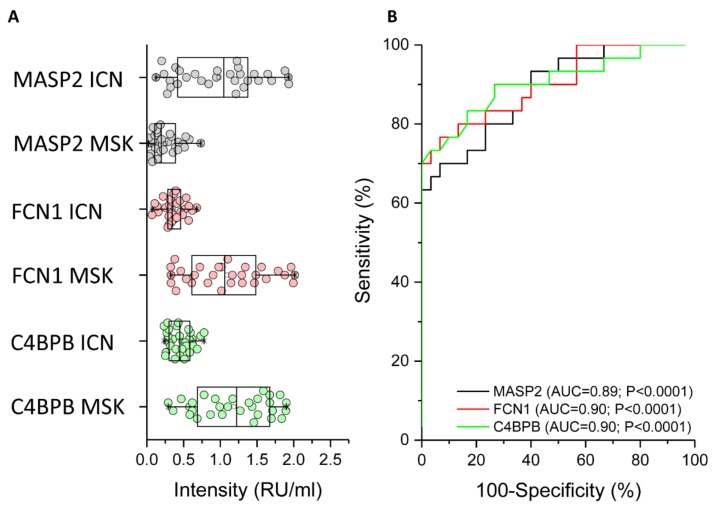
ELISA experiments to verify the proteomic data. (**A**) Box plot showing the median and interquartile range values of MASP2 (black), FCN1 (red), and C4BPB (green) in the urinary exosome of all patients. In particular, MASP2 was strongly expressed in the urinary exosome of ICN compared to MSK patients, whereas FCN1 and C4BPB showed the opposite profile. (**B**) Received operating characteristic (ROC) curve analysis confirming that the expression of all three proteins discriminates between ICN and MSK patients.

The mass spectrometry (MS) results were verified by ELISA for all patients enrolled in the study using an in-house assay setup (Figure 6). We found that MASP2 was expressed more strongly in ICN patients compared to MSK patients (Figure 6A, black circle). The median (and interquartile range) values for ICN and MSK were 1.05 (0.41–1.4) and 0.2 (0.1–0.4), respectively (*p* < 0.0001). Received operating characteristic (ROC) analysis revealed that the expression of MASP2 in the urinary exosome can distinguish between ICN and MSK patients (Figure 6B, black line). The area under the curve (AUC), 95% confidence interval (CI), and p-value for the ROC analysis were 0.89, 0.81–0.97, and *p* < 0.0001, respectively. The cutoff, sensitivity, specificity, and likelihood ratio were 0.62, 67%, 97%, and 20, respectively.

In contrast to MASP2, FCN1 (Figure 6A, red circle) and C4BPB (Figure 6A, green circle) were more strongly expressed in MSK patients compared to ICN patients. For FCN1, the median (and interquartile range) values for ICN and MSK were 0.34 (0.28–0.47) and 1.06 (0.59–1.5), respectively (*p* < 0.0001). ROC analysis revealed that the expression of FCN1 in the urinary exosome can distinguish between ICN and MSK patients (Figure 6B, red line). The AUC, 95% CI and p-values for the ROC analysis were 0.9, 0.82–0.98, and *p* < 0.0001, respectively. The cutoff, sensitivity, specificity, and likelihood ratio were 0.55, 77%, 87%, and 5.7, respectively. For C4BPB, the median (and interquartile range) values for ICN and MSK were 0.45 (0.3–0.59) and 1.2 (0.67–1.67), respectively (*p* < 0.0001). ROC analysis revealed that the expression of C4BPB in the urinary exosome can distinguish between ICN and MSK patients (Figure 6B, green line). The AUC, 95% CI, and p-values for the ROC analysis were 0.9, 0.82–0.98, and *p* < 0.0001, respectively. The cutoff, sensitivity, specificity, and likelihood ratio were 0.62, 77%, 87%, and 5.7, respectively.

## 3. Discussion

MSK is a rare kidney disease that is currently difficult to diagnose because the molecular basis of pathogenesis is unclear and robust diagnostic biomarkers are not available in the clinic. We, therefore, carried out a comprehensive comparative proteomic analysis of urinary microvesicles and exosomes from MSK patients and a control group with the distinct kidney disorder ICN. We defined the protein profiles of both vesicles in both diseases and applied innovative statistical methods (including SVM learning and PLS-DA) to identify specific proteins (and the corresponding biological networks) that can accurately discriminate between patients with each disease. These proteins and networks not only represent a source of novel biomarker candidates and potential therapeutic targets but should also shed light on the molecular basis of MSK.

Statistical analysis identified a core panel of 20 highly discriminative proteins, the most promising were FCN1, MASP2, and C4BPB, all of which are major functional or regulatory components of the lectin complement pathway. These three proteins were not associated with either MSK or ICN according to the DisGeNET, a database containing one of the largest publicly available collections of genes and variants associated with human diseases [27]. FCN1 and C4BPB were more abundant in the exosomes of MSK patients compared to ICN controls, whereas MASP2 showed the opposite profile. It is possible that all three proteins, because of their molecular weight and the absence of proteinuria and/or renal impairment in our enrolled patients, did not pass through an intact glomerular membrane but were instead derived from renal epithelial cells [28].

FCN1 (also known as M-ficolin) is an oligomeric pattern-recognition protein consisting of an N-terminal collagen-like domain and a C-terminal globular fibrinogen-like domain [29,30]. It can form complexes with MASP2 to activate the lectin complement pathway [31]. FCN1 may also interact with C-reactive protein (CRP) via a conformational change induced by mild acidosis at the local site of infection [32,33], and with pentraxin 3 (PTX3) in a Ca^2+^-dependent manner [34]. The high level of FCN1 in urinary exosomes isolated from our MSK patients suggests that the lectin complement pathway may be involved in the complex biological process leading to the growth of cysts. This agrees with previous studies based on mouse models deficient in complement factors, which develop fewer kidney cysts and suffer less severe pericystic tissue inflammation, helping to preserve normal renal functions [35]. Further evidence that the complement pathway is involved in cystic disorders has been gathered from patients affected by ADPKD, whose urine and cyst fluids were found to be enriched for complement proteins [36,37,38].

The downregulation of MASP2 in our MSK patients was accompanied by the upregulation of C4BPB, a multimeric protein that inhibits the activation of the complement cascade by preventing the formation of the classical C3 and C5 convertases [39]. This may reflect the physiological attempt of the kidneys to mitigate the activation of the lectin complement pathway. This could also explain the contra-regulation of FCN1 and MASP2. However, additional studies are necessary to confirm this effect. Indeed, the hyperactivation of complement may induce glomerular and tubulointerstitial injury [40].

Taken together, our data suggest for the first time that the lectin complement pathway may be involved in the complex biological machinery associated with MSK, but it is clearly not associated with nephrolithiasis. Extracellular vesicles may, therefore, be part of the pathological machinery leading to the growth of cysts. Although we cannot exclude that the complement system could be a secondary response to the disease, this might offer a therapeutic option for progression blockade. Additionally, urinary vesicles may provide a source of valuable clinical biomarkers allowing MSK to be distinguished from other cystic disorders (including ADPKD) and types of nephrolithiasis.

However, our results must be validated using a larger cohort of patients, and additional functional studies are required to better define the biological role of the lectin complement cascade in the pathogenesis of MSK.

## 4. Materials and Methods

### 4.1. Patients

Having obtained informed written consent, we enrolled 15 adult MSK patients and 15 adult ICN patients matched for age, gender, and geographical origin, followed up at our renal unit. The inclusion criteria for the MSK group were the same as described in our previous study [12]. Particularly, patients had both kidneys involved, nephrocalcinosis and/or cysts in at least 2 papillae in each kidney. For MSK, patients had papillary precalyceal ectasias on films obtained at least 10 min after contrast medium injection in the absence of compression maneuvers and signs of obstruction. The X-ray films were reviewed by an independent radiologist to confirm the diagnosis. For ICN patients the inclusion criteria were calcium stone disease, normal serum creatinine and electrolyte concentrations, and urinary pH ≤ 5.5 measured in spot morning urine (after overnight fasting) to exclude tubular acidosis. Exclusion criteria for ICN patients were: the presence of endocrine diseases and cystic kidney disorders, nephrocalcinosis, and obstructive nephropathy. In all ICN patients, the MSK disease was ruled out by careful clinical examination.

Clinical data were registered in an electronic database. Informed consent was obtained from all patients in accordance with the Declaration of Helsinki and the ethical board of the University Hospital of Verona approved the study (1312CESC, 24 May 2017).

### 4.2. Isolation of Microvesicles and Exosomes

Second morning urine samples were obtained from all patients. Microvesicles and exosomes were isolated by centrifugation as previously reported [18]. Briefly, 16 ml aliquots of urine were centrifuged at 16,000× *g* for 30 min at 16 °C in order to remove alive and dead cells and organelles. The obtained supernatant was centrifuged for 120 min at 22,000× *g* at 16 °C to obtain the microvesicle fraction. The microvesicle-containing pellet was washed in phosphate-buffered saline (PBS) and repelleted as above for a total of five wash cycles to obtain a clean microvesicle fraction. To obtain the exosome, the supernatant was centrifuged for 120 min at 100,000× *g* at 16 °C. Then one-step sucrose cushion ultracentrifugation for 120 min at 100,000× *g* at 16 °C was performed to further purify exosomes according to their density. The exosome-containing pellet was washed in PBS and repelleted as above for a total of five wash cycles to obtain a clean exosome fraction. This procedure was carried out for each sample and the microvesicle and exosome fractions were stored at –80 °C until use.

### 4.3. Dynamic Light Scattering

The size of the exosomes and microvesicles was determined by dynamic light scattering (DLS) using a Zetasizer nano ZS90 particle sizer (Malvern Instruments, Worcestershire, UK) at a 90° fixed angle. The particle diameter was calculated using the Stokes–Einstein equation. For particle sizing in solution, exosome and microvesicle aliquots were diluted in 10% PBS and analyzed at a constant 25 °C.

### 4.4. Western Blot

Microvesicle and exosome fractions from 16 ml urine samples were separated by SDS-PAGE (8–16% acrylamide gradient) and then transferred to a nitrocellulose membrane. The membrane was blocked with 1% bovine serum albumin (BSA) in PBS plus 0.1% Tween-20 (PBST), rinsed in PBST, and labeled with one of the following primary human antibodies diluted in 1% BSA in PBST: anti-CD63 (Novus Biological, Littleton, CA, USA), anti-CD81 (Novus Biological, Littleton, CA, USA), or anti-CD45 (LifeSpan BioSciences, Seattle, WA, USA) [41]. After rinsing again in PBST, the membrane was incubated with horseradish peroxidase (HRP)-conjugated anti-mouse secondary antibodies (Novus Biological, Littleton, CA, USA). Chemiluminescence was detected and quantified using the ChemiDoc Touch Imaging System and Image Lab software (Bio-Rad, Hercules, CA, USA).

### 4.5. Mass Spectrometry

Samples were processed with in-StageTip (iST) method with two poly (styrene divinylbenzene) reversed-phase sulfonate (SDB-RPS) disks. The microvesicle- and exosome-containing pellets were solubilized with a solution containing 10 mM Tris(2-carboxyethyl)phosphine, 40 mM chloro-acetamide, 100 mM Tris (pH 8.5), and 2% sodium deoxycholate. Lysis, reduction, and alkylation of the samples were performed in a single step and then loaded into the StageTip. The samples were then diluted with a buffer containing 25 mM Tris (pH 8.5) and 1 µg of trypsin. After the acidification step with 1% trifluoroacetic acid (TFA), the samples were washed with 0.2% TFA three times. The proteins were eluted in 60 µl 5% v/v ammonium hydroxide containing 80% v/v acetonitrile. The desalted peptides were dried in a speed vacuum and resuspended in 2% acetonitrile containing 0.2% formic acid (FA). They were then separated on a 50-cm reversed-phase Easy Spray column (75-µm internal diameter × 50 cm; 2 μm/100 Å C18) on an Ultimate 3000 RSLCnano device (Thermo Fisher Scientific, Waltham, MA, USA) with a binary buffer system comprising buffer A (0.1% FA) and buffer B (80% acetonitrile, 5% dimethylsulfoxide, 0.1% FA). The program consisted of a 70-min gradient (2–45% buffer B) at a flow rate of 250 μl/min, with the column temperature maintained at 60 °C. The chromatography system was coupled to an Orbitrap Fusion Tribrid mass spectrometer (Thermo Fisher Scientific, Waltham, MA, USA), acquiring data in Charge Ordered Parallel Ion aNalysis (CHOPIN) mode. The precursors were ionized using an EASY-spray source held at +2.2 kV and the iICNet capillary temperature was held at 300 °C. Single MS survey scans were performed over the mass window 375–1500 *m/z* with an AGC target of 250,000, a maximum injection time of 50 ms, and a resolution of 120,000 at 200 *m/z*. Monoisotopic precursor selection was enabled for peptide isotopic distributions, precursors of z = 2–5 were selected for 2 s of cycle time, and dynamic exclusion was set to 25 s with a ± 10 ppm window set around the precursor. The following CHOPIN conditions were applied: a) if the precursor charge state is 2, then follow with collision-induced dissociation (CID) and scan in the ion trap with an isolation window of 1.8, CID energy of 35%, and a rapid ion trap scan rate; b) if the precursor charge state is 3–5 and precursor intensity >500,000, then follow with higher-energy C-trap dissociation (HCD) and scan in the Orbitrap with an isolation window of 1.8, HCD energy of 28%, and a resolution of 15,000; c) if the precursor charge state is 3–5 and precursor intensity < 500,000, then follow with CID as described for option (a). For all MS^2^ events, the following options were set: “Injection Ions for All Available Parallelizable Time” with an AGC target value of 4000 and a maximum injection time of 250 ms for CID, or an AGC target value of 10,000 and a maximum injection time of 40 ms for HCD.

### 4.6. MS Data Analysis

Raw MS files were processed in the MaxQuant v1.6.0.16 environment using the MaxLFQ algorithm for label-free quantification and Andromeda search engine. Peptide and protein false discovery rate (FDRs) were both set at < 0.01. The search contained variable modifications for the oxidation of methionine (M), N-terminal protein acetylation (protein N-terminus)*,* and fixed carbamidomethyl modifications (C). For protease digestion, up to two missed cleavages were allowed. Peptides of length greater than six amino acids have been considered for the identification. The “match between runs” algorithm with a setting of 1 min time window was used for the quantification of MS missed data in each individual measurement. UniProt FASTA Homo sapiens database (access August 2017) was used for the identification of peptides and proteins.

### 4.7. ELISA

MASP2, FNC1, and C4BPB levels were determined using an in-house ELISA setup. Briefly, polyclonal antibody anti-MASP2, anti-FCN1, or anti-C4BPB (LifeSpan Biosciences) was used to coat overnight at 4 °C 96-well MaxiSorp Nunc plates (Thermo Fisher Scientific, Waltham, MA, USA). The plates were then blocked with 3% BSA in PBS. Exosome fractions were solubilized in 10 µl of mild detergent solution (1% Nonidet P-40, 0.5% Tween-20 in PBS), supplemented with 90 µl 3% BSA in PBST and incubated at 4 °C overnight. After three washes in PBST, the plates were incubated for 4h with the corresponding monoclonal antibody (anti-MASP2, anti-FCN1, or anti-C4BPB, all from LifeSpan Biosciences, Seattle, WA, USA) diluted 1:1000 with 1% BSA in PBST. After three washes with PBST, the plates were incubated with HRP-conjugated anti-mouse IgG diluted 1:5000 in 1% BSA in PBST for 1h. After washing, the peroxidase substrate (TMB, Bio-Rad, Hercules, CA, USA) was added. The reaction was stopped with an H_2_SO_4_ solution. The absorbance at 450 nm was measured using an iMark microplate reader (Bio-Rad, Hercules, CA, USA). To standardize the response of the antibodies, we used a pool of strongly positive controls. The optical density results were expressed as relative units per milliliter (RU/ml).

### 4.8. Statistical Analysis

After normalization, unsupervised hierarchical clustering and multidimensional scaling (MDS) with Spearman’s rank correlation and *k*-means were used to recognize outliers and sample dissimilarity. Weighted-gene co-expression network analysis (WGCNA) package in *R* was used for building the co-expression network with the normalized expression profiles of the identified proteins. A weighted adjacency matrix was constructed using the power function. After choosing the appropriate beta parameter of power (with the value of independence scale set to 0.8), the adjacency matrix was transformed into a topological overlap matrix (TOM), which measures the network connectivity of all the proteins. To classify proteins with co-expression profiles into protein modules, hierarchical clustering analysis was conducted according to the TOM dissimilarity with a minimum size of 30 proteins per module. To identify the relationship between each module and clinical trait, we used module eigengenes (MEs) and calculated the correlation between MEs and each clinical trait and their statistical significance corrected for multiple interactions. A heat map was then used to visualize the degree and significance of each relationship.

A non-parametric Mann–Whitney *U*-test, machine learning methods, such as non-linear support vector machine (SVM) learning, and partial least squares discriminant analysis (PLS-DA) were used to identify the hub proteins of modules that maximize the discrimination between the selected clinical traits. For the Mann–Whitney *U*-test, proteins were considered to be significantly differentially expressed between the two conditions with power of 80% and an adjusted p-value ≤ 0.05 after correction for multiple interactions (Benjamini–Hochberg) and a fold change of ≥2. In addition, the proteins needed to show at least 70% identity in the samples in one of two conditions. Volcano plots were used to visualize this analysis. In SVM learning, a fourfold cross-validation approach was applied to estimate the prediction and classification accuracy. The whole matrix was also randomly divided into two parts, one for learning (65%) and the other to verify the accuracy of the prediction (35%). Finally, the resulting core panel of hub proteins was uploaded to Cytoscape using the EnrichmentMap, ClusterMaker2, and AutoAnnotate apps to construct a protein–protein interaction network and identify the principal biological processes and pathways involved in each condition. Gene Ontology (GO) annotations were extracted from the Gene Ontology Consortium (http://www.geneontology.org/).

For ELISA data analysis, the Mann–Whitney *U*-test was used to assess differences in MASP2, FCN1, or C4BPB protein levels between the two study groups, and the results were expressed as medians and interquartile ranges. A value of *p* < 0.05 was considered to be statistically significant. Received operating characteristic (ROC) curves were generated to assess the diagnostic efficiency of each assay. Youden’s index and likelihood ratio were used, to identify the cutoff and the diagnostic performance of the tests, respectively. All statistical tests were performed using the latest version of the software package *R* available at the time of the experiments.

## Supplementary Materials

Supplementary materials can be found at https://www.mdpi.com/1422-0067/20/21/5517/s1. Figure S1. Characterization of exosome and microvesicle fractions purified from the urine samples of ICN and MSK patients. Figure S2. Venn diagram of all proteins identified in exosomes and microvesicles isolated from the urine of ICN and MSK patients, and proteins associated with ICN and MSK. Figure S3. Multidimensional scaling analysis of extracellular vesicles from the urine of ICN and MSK patients. Figure S4. Exosome and microvesicle proteins that show significant differences in abundance between ICN and MSK patients. Figure S5. Enrichment network map of the significant proteins found in urinary microvesicles and exosomes from MSK and ICN patients. Table S1. List of identification values for all significant proteins between MSK and ICN in exosome and microvesicle fraction. Table S2. List of all significant proteins between MSK and ICN in exosome and microvesicle fraction.

## Abbreviations

MSK	medullary sponge kidney
ICN	idiopathic calcium nephrolithiasis
LAMA2	laminin subunit α2
SVM	support vector machine
PLS-DA	partial least squares discriminant analysis
ADPKD	autosomal dominant polycystic kidney disease
MS	mass spectrometry
SPP1	osteopontin
PBS	phosphate-buffered saline
DLS	dynamic light scattering
iST	in-StageTip
SDB-RPS	poly (styrene divinylbenzene) reversed-phase sulfonate
TFA	trifluoroacetic acid
BSA	bovine serum albumin
PBST	PBS plus 0.1% Tween-20
HRP	horseradish peroxidase
ROC	Received operating characteristic
AUC	area under the curve
CI	confidence interval
FCN1	Ficolin 1
MASP2	Mannan-binding lectin serine protease 2
C4BPB	Complement component 4-binding protein β
PTX3	pentraxin 3
CRP	C-reactive protein
