# Supplementary material for: Proteomic Analysis of Urinary Extracellular Vesicles Reveals a Role for the Complement System in Medullary Sponge Kidney Disease
Source: Int J Mol Sci. 2019 Nov 5;20(21):5517. doi: 10.3390/ijms20215517 (PMC6862015; doi:10.3390/ijms20215517)
Supplement: Supplementary file 1 [file ijms-20-05517-s001.zip › Bruschi et al_Supplementary Table 1.docx]

**Supplemental Table 1.** List of identification values for all discriminatory proteins between MSK and ICN in exosome and microvesicle fraction.

| Uniprot IDs | Protein names | Gene name | Peptides | Unique peptides | Sequence coverage [%] | Unique  sequence coverage [%] | Score |
| --- | --- | --- | --- | --- | --- | --- | --- |
| Q5H8C1 | FRAS1-related extracellular matrix protein 1 | FREM1 | 14 | 14 | 9.9 | 9.9 | 38.44 |
| P49411 | Elongation factor Tu, mitochondrial | TUFM | 3 | 3 | 4.9 | 4.9 | 3.59 |
| P50135 | Histamine N-methyltransferase | HNMT | 6 | 6 | 23.3 | 23.3 | 14.08 |
| P48764-2 | Sodium/hydrogen exchanger 3 | SLC9A3 | 8 | 8 | 14.7 | 14.7 | 59.6 |
| P30038 | Delta-1-pyrroline-5-carboxylate dehydrogenase, mitochondrial | ALDH4A1 | 2 | 2 | 8 | 8 | 4.6 |
| Q9BSW2-2 | EF-hand calcium-binding domain-containing protein 4B | CRACR2A | 10 | 10 | 20.5 | 20.5 | 13.33 |
| Q5VZK9-2 | Leucine-rich repeat-containing protein 16A | LRRC16A | 17 | 17 | 20.3 | 20.3 | 28.41 |
| Q9BVM4 | Gamma-glutamylaminecyclotransferase | GGACT | 4 | 4 | 40.5 | 40.5 | 55.99 |
| P13716 | Delta-aminolevulinic acid dehydratase | ALAD | 9 | 9 | 40.9 | 40.9 | 93.24 |
| Q9Y3R5-2 | Protein dopey-2 | DOPEY2 | 39 | 39 | 25.6 | 25.6 | 118.79 |
| A0A087X0T8 | Cell adhesion molecule 1 | CADM1 | 3 | 3 | 13 | 13 | 6.16 |
| Q99969 | Retinoic acid receptor responder protein 2 | RARRES2 | 4 | 4 | 38.7 | 38.7 | 42.79 |
| G3XAE9 | Protein FAM179B | FAM179B | 21 | 21 | 19.1 | 19.1 | 36.55 |
| Q9Y2V2 | Calcium-regulated heat stable protein 1 | CARHSP1 | 4 | 4 | 48.3 | 48.3 | 8.49 |
| A0A087X1K9 | Acyl-protein thioesterase 1 | LYPLA1 | 4 | 1 | 38.6 | 15.7 | 5.74 |
| O95834 | Echinoderm microtubule-associated protein-like 2 | EML2 | 10 | 9 | 18.8 | 17.6 | 41.36 |
| P11586 | C-1-tetrahydrofolate synthase, cytoplasmic | MTHFD1 | 18 | 1 | 21.5 | 1.3 | 32.32 |
| K7ES70 | Microfibril-associated glycoprotein 4 | MFAP4 | 5 | 5 | 27.5 | 27.5 | 34.99 |
| I3L0N3 | Vesicle-fusing ATPase | NSF | 16 | 16 | 31 | 31 | 51.69 |
| P31153-2 | S-adenosylmethionine synthase isoform type-2 | MAT2A | 3 | 3 | 13 | 13 | 6.62 |
| E9PGM4 | 1,4-alpha-glucan-branching enzyme | GBE1 | 16 | 16 | 31.5 | 31.5 | 42.26 |
| Q14117 | Dihydropyrimidinase | DPYS | 9 | 8 | 22.7 | 21.4 | 48.44 |
| P50990-2 | T-complex protein 1 subunit theta | CCT8 | 11 | 11 | 24.2 | 24.2 | 20.56 |
| C9JA99 | Protocadherin alpha-13 | PCDHA13 | 6 | 2 | 8.4 | 1.9 | 52.93 |
| P27449 | V-type proton ATPase 16 kDa proteolipid subunit | ATP6V0C | 4 | 3 | 36.1 | 31.6 | 20.64 |
| O95398-3 | Rap guanine nucleotide exchange factor 3 | RAPGEF3 | 13 | 13 | 25.8 | 25.8 | 18.89 |
| O15197 | Ephrin type-B receptor 6 | EPHB6 | 5 | 5 | 7.5 | 7.5 | 15.29 |
| P30613-2 | Pyruvate kinase PKLR | PKLR | 12 | 11 | 33.1 | 31.1 | 66.02 |
| O96013 | Serine/threonine-protein kinase PAK 4 | PAK4 | 9 | 9 | 18.8 | 18.8 | 162.77 |
| E7EX90 | Dynactin subunit 1 | DCTN1 | 7 | 7 | 7.4 | 7.4 | 9.07 |
| Q9NQE9 | Histidine triad nucleotide-binding protein 3 | HINT3 | 4 | 4 | 29.7 | 29.7 | 15.95 |
| O94919 | Endonuclease domain-containing 1 protein | ENDOD1 | 8 | 8 | 24.4 | 24.4 | 63.84 |
| F8WC39 | Glycerol kinase | GK | 5 | 5 | 57.8 | 57.8 | 7.95 |
| A0A096LP62 | Inter-alpha-trypsin inhibitor heavy chain H5 | ITIH5 | 5 | 5 | 8.8 | 8.8 | 13.06 |
| E7EVJ5 | Cytoplasmic FMR1-interacting protein 2 | CYFIP2 | 18 | 7 | 18.1 | 6.8 | 44.13 |
| Q96BW5-2 | Phosphotriesterase-related protein | PTER | 10 | 10 | 41.7 | 41.7 | 91.69 |
| P15907 | Beta-galactoside alpha-2,6-sialyltransferase 1 | ST6GAL1 | 7 | 7 | 23.6 | 23.6 | 18.7 |
| Q9Y2E5 | Epididymis-specific alpha-mannosidase | MAN2B2 | 22 | 22 | 33.6 | 33.6 | 201.26 |
| O60637-3 | Tetraspanin-3 | TSPAN3 | 3 | 3 | 15.9 | 15.9 | 5.76 |
| Q02790 | Peptidyl-prolyl cis-trans isomerase FKBP4 | FKBP4 | 11 | 11 | 30.1 | 30.1 | 42.74 |
| P12955 | Xaa-Pro dipeptidase | PEPD | 15 | 15 | 38.5 | 38.5 | 211.58 |
| E7EVZ1 | Zinc finger homeobox protein 4 | ZFHX4 | 3 | 3 | 0.7 | 0.7 | 2.9 |
| D6R9I9 | ATP-binding cassette sub-family E member 1 | ABCE1 | 1 | 1 | 2.6 | 2.6 | 2 |
| E5RHG8 | Transcription elongation factor B polypeptide 1 | TCEB1 | 3 | 3 | 47.2 | 47.2 | 5.33 |
| Q9H993 | Protein-glutamate O-methyltransferase | ARMT1 | 8 | 8 | 28.8 | 28.8 | 29.25 |
| D6RDU5 | Septin-11 | SEPT11 | 5 | 5 | 21.8 | 21.8 | 11.36 |
| P31689 | DnaJ homolog subfamily A member 1 | DNAJA1 | 10 | 10 | 40.8 | 40.8 | 75.06 |
| H3BPK3 | Hydroxyacylglutathione hydrolase, mitochondrial | HAGH | 4 | 4 | 20.5 | 20.5 | 5.85 |
| P36507 | Dual specificity mitogen-activated protein kinase kinase 2 | MAP2K2 | 6 | 6 | 30 | 30 | 17.01 |
| O94903 | Proline synthase co-transcribed bacterial homolog protein | PROSC | 4 | 4 | 20.4 | 20.4 | 5.41 |
| P49189 | 4-trimethylaminobutyraldehyde dehydrogenase | ALDH9A1 | 9 | 9 | 23.1 | 23.1 | 22.93 |
| Q15848 | Adiponectin | ADIPOQ | 3 | 3 | 21.7 | 21.7 | 121.69 |
| Q9UHI8 | A disintegrin and metalloproteinase with thrombospondin motifs 1 | ADAMTS1 | 2 | 2 | 5.3 | 5.3 | 129.45 |
| Q58FF8 | Putative heat shock protein HSP 90-beta 2 | HSP90AB2P | 8 | 2 | 21.8 | 7.1 | 6.16 |
| P09619 | Platelet-derived growth factor receptor beta | PDGFRB | 5 | 5 | 6.2 | 6.2 | 10.32 |
| Q9BZV1 | UBX domain-containing protein 6 | UBXN6 | 11 | 11 | 40.8 | 40.8 | 47.42 |
| P40189-3 | Interleukin-6 receptor subunit beta | IL6ST | 9 | 9 | 11.4 | 11.4 | 153.48 |
| E9PP76 | Superoxide dismutase [Cu-Zn] | CCS | 3 | 3 | 42.6 | 42.6 | 3.94 |
| P17252 | Protein kinase C alpha type | PRKCA | 5 | 5 | 8.8 | 8.8 | 10.26 |
| P21709-3 | Ephrin type-A receptor 1 | EPHA1 | 4 | 4 | 9.3 | 9.3 | 44.16 |
| Q14376 | UDP-glucose 4-epimerase | GALE | 8 | 8 | 36.2 | 36.2 | 33.02 |
| G3V3U4 | Proteasome subunit alpha type | PSMA6 | 3 | 3 | 32.7 | 32.7 | 8.6 |
| Q9BTY2 | Plasma alpha-L-fucosidase | FUCA2 | 15 | 15 | 45.8 | 45.8 | 181.42 |
| O15031 | Plexin-B2 | PLXNB2 | 14 | 14 | 12.2 | 12.2 | 57.42 |
| P49588 | Alanine--tRNA ligase, cytoplasmic | AARS | 11 | 11 | 17.1 | 17.1 | 25.5 |
| D6RB59 | Exocyst complex component 3 | EXOC3 | 2 | 2 | 5.6 | 5.6 | 3.54 |
| P00167-2 | Cytochrome b5 | CYB5A | 2 | 2 | 27.6 | 27.6 | 4.14 |
| E9PIR7 | Thioredoxin reductase 1, cytoplasmic | TXNRD1 | 6 | 6 | 19.7 | 19.7 | 6.32 |
| Q92887 | Canalicular multispecific organic anion transporter 1 | ABCC2 | 6 | 6 | 4.8 | 4.8 | 6.14 |
| A0A087X0R6 | Sorting nexin-12 | SNX12 | 6 | 4 | 47.7 | 38.4 | 7.53 |
| Q9HAT8 | E3 ubiquitin-protein ligase pellino homolog 2 | PELI2 | 3 | 3 | 7.6 | 7.6 | 3.85 |
| A0A087WVM2 | CD177 antigen | CD177 | 3 | 3 | 6.6 | 6.6 | 15.82 |
| P28074 | Proteasome subunit beta type-5 | PSMB5 | 4 | 4 | 18.6 | 18.6 | 6.35 |
| A0A0A0MSE9 | Pleckstrin homology domain-containing family B member 2 | PLEKHB2 | 3 | 3 | 14.4 | 14.4 | 15.82 |
| P28072 | Proteasome subunit beta type-6 | PSMB6 | 4 | 4 | 29.3 | 29.3 | 10.18 |
| P82980 | Retinol-binding protein 5 | RBP5 | 5 | 5 | 45.2 | 45.2 | 9.81 |
| P11908 | Ribose-phosphate pyrophosphokinase 2 | PRPS2 | 3 | 3 | 14.8 | 14.8 | 9.96 |
| F8W810 | Putative eukaryotic translation initiation factor 2 subunit 3-like protein | EIF2S3L | 1 | 1 | 5.4 | 5.4 | 6.21 |
| P0C0L4 | Complement C4-A | C4A | 91 | 1 | 61.9 | 0.7 | 323.31 |
| E7END6 | Vitamin K-dependent protein C | PROC | 4 | 4 | 10.7 | 10.7 | 13.08 |
| A0A1W2PQB1 | Low affinity immunoglobulin gamma Fc region receptor III-A | FCGR3A | 6 | 2 | 17.6 | 7.9 | 58.12 |
| A6QRJ0 | Deoxyribonuclease-1-like 1 | DNASE1L1 | 3 | 3 | 26 | 26 | 16.13 |
| P17213 | Bactericidal permeability-increasing protein | BPI | 12 | 12 | 42.3 | 42.3 | 323.31 |
| P02743 | Serum amyloid P-component | APCS | 8 | 8 | 38.1 | 38.1 | 69.73 |
| B1AMW1 | Lymphocyte function-associated antigen 3 | CD58 | 2 | 2 | 7.5 | 7.5 | 6.63 |
| Q96KN2 | Beta-Ala-His dipeptidase | CNDP1 | 12 | 12 | 30.8 | 30.8 | 282.58 |
| P02679 | Fibrinogen gamma chain | FGG | 32 | 22 | 80.4 | 58.7 | 323.31 |
| P02675 | Fibrinogen beta chain | FGB | 31 | 25 | 62.7 | 55.4 | 323.31 |
| P07360 | Complement component C8 gamma chain | C8G | 4 | 4 | 29.7 | 29.7 | 135.03 |
| P00739 | Haptoglobin-related protein | HPR | 25 | 9 | 61.8 | 33.6 | 212.54 |
| V9GYM3 | Apolipoprotein A-II | APOA2 | 7 | 7 | 48.1 | 48.1 | 323.31 |
| P03951-2 | Coagulation factor XI | F11 | 11 | 11 | 22.4 | 22.4 | 50.08 |
| P27701-2 | CD82 antigen | CD82 | 5 | 5 | 27.7 | 27.7 | 19.92 |
| Q9Z2K1 |  | KRT16 | 11 | 1 | 21.5 | 1.5 | 2 |
| P01624 | Ig kappa chain V-III region POM | IGKV3OR2-268 | 2 | 1 | 20.9 | 7.8 | 2 |
| Q8ND23-3 | Capping protein, Arp2/3 and myosin-I linker protein 3 | CARMIL3 | 1 | 1 | 11.6 | 11.6 | 2 |
| C9JP03 | SLAIN motif-containing protein 1 | SLAIN1 | 1 | 1 | 16.2 | 16.2 | 2 |
| P04114 | Apolipoprotein B-100 | APOB | 232 | 232 | 64.2 | 64.2 | 323.31 |
| I3L3J2 | Zinc finger and SCAN domain-containing protein 32 | ZSCAN32 | 1 | 1 | 7.3 | 7.3 | 2.6 |
| Q9H9A6 | Leucine-rich repeat-containing protein 40 | LRRC40 | 3 | 3 | 7.6 | 7.6 | 3.18 |
| Q8N122-3 | Regulatory-associated protein of mTOR | RPTOR | 2 | 2 | 2.5 | 2.5 | 3.55 |
| C9JD53 | Isopentenyl-diphosphate Delta-isomerase 1 | IDI1 | 2 | 2 | 15 | 15 | 3.44 |
| Q02487-2 | Desmocollin-2 | DSC2 | 13 | 12 | 20.1 | 19 | 27.28 |
| P07225 | Vitamin K-dependent protein S | PROS1 | 13 | 11 | 23.5 | 21 | 156.64 |
| P04003 | C4b-binding protein alpha chain | C4BPA | 10 | 10 | 20.8 | 20.8 | 52.94 |
| Q5S007 | Leucine-rich repeat serine/threonine-protein kinase 2 | LRRK2 | 13 | 13 | 7.7 | 7.7 | 26.4 |
| Q6NXT6-2 | Transmembrane anterior posterior transformation protein 1 homolog | TAPT1 | 2 | 2 | 6.8 | 6.8 | 2 |
| Q9UJU2-4 | Lymphoid enhancer-binding factor 1 | LEF1 | 1 | 1 | 10.3 | 10.3 | 2 |
| A4FU69-2 | EF-hand calcium-binding domain-containing protein 5 | EFCAB5 | 4 | 4 | 5 | 5 | 5 |
| A0A0C4DG40 | Nesprin-1 | SYNE1 | 6 | 6 | 0.8 | 0.8 | 3.2 |
| Q8NDA2-2 | Hemicentin-2 | HMCN2 | 6 | 6 | 1.7 | 1.7 | 48.66 |
| P55291 | Cadherin-15 | CDH15 | 16 | 16 | 27.3 | 27.3 | 74.44 |
| P11678 | Eosinophil peroxidase | EPX | 10 | 7 | 14.8 | 10.8 | 5.66 |
| A0A075B7D9 | RNA-binding protein FUS | TAF15 | 2 | 2 | 5.3 | 5.3 | 3.73 |
| O00182-3 | Galectin-9 | LGALS9 | 7 | 7 | 29.9 | 29.9 | 181.98 |
| Q13733 | Sodium/potassium-transporting ATPase subunit alpha-4 | ATP1A4 | 8 | 1 | 9.4 | 0.8 | 2 |
| Q08554-2 | Desmocollin-1 | DSC1 | 5 | 5 | 7 | 7 | 12.72 |
| Q9UKM7 | Endoplasmic reticulum mannosyl-oligosaccharide 1,2-alpha-mannosidase | MAN1B1 | 12 | 12 | 29 | 29 | 70.96 |
| A0A024R412 | Neuropilin-2 | NRP2 | 2 | 2 | 3 | 3 | 2.87 |
| Q9NSK0 | Kinesin light chain 4 | KLC4 | 5 | 5 | 11.3 | 11.3 | 12.07 |
| Q9NQX4 | Unconventional myosin-Vc | MYO5C | 19 | 17 | 13.4 | 11.9 | 31.55 |
| O00187-2 | Mannan-binding lectin serine protease 2 | MASP2 | 8 | 8 | 49.7 | 49.7 | 323.31 |
| P08572 | Collagen alpha-2(IV) chain | COL4A2 | 14 | 14 | 11.9 | 11.9 | 207.06 |
| Q6UWV6 | Ectonucleotide pyrophosphatase/phosphodiesterase family member 7 | ENPP7 | 10 | 9 | 33.8 | 31.7 | 119.24 |
| X6R868 | Carboxylic ester hydrolase | CEL | 22 | 22 | 40.7 | 40.7 | 323.31 |
| P41181 | Aquaporin-2 | AQP2 | 6 | 6 | 35.4 | 35.4 | 261.61 |
| P20160 | Azurocidin | AZU1 | 3 | 3 | 12 | 12 | 4.6 |
| Q16769 | Glutaminyl-peptide cyclotransferase | QPCT | 17 | 17 | 71.5 | 71.5 | 323.31 |
| P51151 | Ras-related protein Rab-9A | RAB9A | 3 | 3 | 19.9 | 19.9 | 20.79 |
| Q9UKU9 | Angiopoietin-related protein 2 | ANGPTL2 | 11 | 9 | 27.2 | 22.1 | 323.31 |
| P31371 | Fibroblast growth factor 9 | FGF9 | 5 | 5 | 28.8 | 28.8 | 150.37 |
| P22894 | Neutrophil collagenase | MMP8 | 9 | 9 | 26.6 | 26.6 | 91.96 |
| P15104 | Glutamine synthetase | GLUL | 2 | 2 | 7.5 | 7.5 | 92.34 |
| P20851-2 | C4b-binding protein beta chain | C4BPB | 3 | 3 | 12 | 12 | 2.79 |
| O00602 | Ficolin-1 | FCN1 | 3 | 3 | 16 | 16 | 24.48 |
| A8MTJ3 | Guanine nucleotide-binding protein G(t) subunit alpha-3 | GNAT3 | 3 | 1 | 7.1 | 1.7 | 2 |
| A0A0A0MRJ7 | Coagulation factor V | F5 | 19 | 16 | 11.2 | 9.7 | 189.78 |
| J3KNB4 | Cathelicidin antimicrobial peptide | CAMP | 4 | 4 | 22 | 22 | 51.06 |
| A0A087WUZ3 | Spectrin beta chain, non-erythrocytic 1 | SPTBN1 | 15 | 15 | 9.2 | 9.2 | 105.28 |
| P07237 | Protein disulfide-isomerase | P4HB | 12 | 12 | 28.3 | 28.3 | 79.81 |
| P28070 | Proteasome subunit beta type-4 | PSMB4 | 6 | 6 | 36.4 | 36.4 | 21.63 |
| O14791 | Apolipoprotein L1 | APOL1 | 17 | 17 | 55.3 | 55.3 | 115.02 |
