# Supplementary material for: Proteomic Analysis of Urinary Extracellular Vesicles Reveals a Role for the Complement System in Medullary Sponge Kidney Disease
Source: Int J Mol Sci. 2019 Nov 5;20(21):5517. doi: 10.3390/ijms20215517 (PMC6862015; doi:10.3390/ijms20215517)
Supplement: Supplementary file 1 [file ijms-20-05517-s001.zip › Bruschi et al_Supplementary Table 2.docx]

**Supplemental Table 2.** List of all discriminatory proteins between MSK and ICN in exosome and microvesicle fraction. The symbol “+” indicates the presence in the different groups of samples or the statistical significance. The P-value is reported only for the proteins with the 70% of presence in at least one groups.

| Uniprot ID | Protein names | Gene name | Log_2_ Fold Change Exosome MSK-NL | -Log_10_ P-value Exosome MSK-NL | Log_2_ Fold Change Microvesicle MSK-NL | -Log_10_ P-value Microvesicle MSK-NL | Presence Exosome MSK | Presence Exosome NL | Presence Microvesicle MSK | Presence Microvesicle NL | Significant Exosome MSK-NL | Significant Microvesicle MSK-NL | ME Color |
| --- | --- | --- | --- | --- | --- | --- | --- | --- | --- | --- | --- | --- | --- |
| Q5H8C1 | FRAS1-related extracellular matrix protein 1 | FREM1 | 3.52 ±1.33 | 1.36 | -8.41 ±0.27 | 15.96 | + | + |  | + |  | + | grey |
| P49411 | Elongation factor Tu, mitochondrial | TUFM | -1.36 ±1.14 | 0.66 | -6.36 ±0.18 | 15.09 | + | + |  | + |  | + | purple |
| P50135 | Histamine N-methyltransferase | HNMT | -2.32 ±0.87 | 1.91 | -4.9 ±0.06 | 14.16 | + | + |  | + |  | + | turquoise |
| P48764-2 | Sodium/hydrogen exchanger 3 | SLC9A3 | -2.81 ±1.63 |  | -4.69 ±0.08 | 5.19 |  | + | + | + |  | + | turquoise |
| P30038 | Delta-1-pyrroline-5-carboxylate dehydrogenase, mitochondrial | ALDH4A1 | 0.15 ±0.56 |  | -4.66 ±0.31 | 11.34 |  | + |  | + |  | + | grey |
| Q9BSW2-2 | EF-hand calcium-binding domain-containing protein 4B | CRACR2A | -1.93 ±0.29 |  | -4.57 ±1.28 | 5.25 | + | + | + | + |  | + | turquoise |
| Q5VZK9-2 | Leucine-rich repeat-containing protein 16A | LRRC16A | 1.08 ±1.21 |  | -3.79 ±1.91 | 4.48 | + | + |  | + |  | + | turquoise |
| Q9BVM4 | Gamma-glutamylaminecyclotransferase | GGACT | -2.28 ±0.33 | 1.87 | -3.61 ±1.32 | 4.82 | + | + |  | + |  | + | brown |
| P13716 | Delta-aminolevulinic acid dehydratase | ALAD | 0.02 ±0.1 | 0.01 | -3.53 ±1.31 | 5.21 | + | + |  | + |  | + | blue |
| Q9Y3R5-2 | Protein dopey-2 | DOPEY2 | -1.11 ±0.26 | 0.72 | -3.52 ±0.14 | 3.47 | + | + | + | + |  | + | blue |
| A0A087X0T8 | Cell adhesion molecule 1 | CADM1 | -1.79 ±1.4 |  | -3.47 ±0.85 | 6.82 |  | + |  | + |  | + | tan |
| Q99969 | Retinoic acid receptor responder protein 2 | RARRES2 | 0.69 ±0.7 |  | -3.46 ±1.31 | 4.74 | + |  |  | + |  | + | red |
| G3XAE9 | Protein FAM179B | FAM179B | -0.8 ±0.43 |  | -3.45 ±0.86 | 6.25 | + | + |  | + |  | + | turquoise |
| Q9Y2V2 | Calcium-regulated heat stable protein 1 | CARHSP1 | -0.96 ±0.32 |  | -3.43 ±1.24 | 4.69 | + | + |  | + |  | + | turquoise |
| A0A087X1K9 | Acyl-protein thioesterase 1 | LYPLA1 | -0.68 ±0.27 | 0.44 | -3.28 ±1.82 | 3.75 | + | + |  | + |  | + | blue |
| O95834 | Echinoderm microtubule-associated protein-like 2 | EML2 | -1.48 ±0.36 | 1.3 | -3.14 ±1.14 | 3.28 | + | + | + | + |  | + | turquoise |
| P11586 | C-1-tetrahydrofolate synthase, cytoplasmic | MTHFD1 | -2.35 ±0.15 | 2.3 | -3.13 ±1.19 | 2.94 | + | + | + | + |  | + | turquoise |
| K7ES70 | Microfibril-associated glycoprotein 4 | MFAP4 | -1.78 ±0.78 | 1.12 | -3.12 ±1.2 | 4.08 | + | + |  | + |  | + | brown |
| I3L0N3 | Vesicle-fusing ATPase | NSF | -0.81 ±0.09 |  | -3.12 ±1.2 | 3.95 | + | + |  | + |  | + | turquoise |
| P31153-2 | S-adenosylmethionine synthase isoform type-2 | MAT2A | -0.43 ±0.06 |  | -3.11 ±1.04 | 5.63 | + | + |  | + |  | + | blue |
| E9PGM4 | 1,4-alpha-glucan-branching enzyme | GBE1 | -1.46 ±0.17 |  | -3.07 ±1.11 | 2.86 | + | + | + | + |  | + | blue |
| Q14117 | Dihydropyrimidinase | DPYS | -1.05 ±0.12 |  | -3.05 ±0.11 | 3.05 | + | + | + | + |  | + | blue |
| P50990-2 | T-complex protein 1 subunit theta | CCT8 | 1.85 ±0.14 | 1.25 | -2.95 ±0.6 | 4.19 | + | + | + | + |  | + | blue |
| C9JA99 | Protocadherin alpha-13 | PCDHA13 | -1.74 ±1.57 | 1.76 | -2.95 ±0.33 | 4.47 | + | + | + | + |  | + | turquoise |
| P27449 | V-type proton ATPase 16 kDa proteolipid subunit | ATP6V0C | -1.56 ±0.6 |  | -2.95 ±1.63 | 3.39 |  | + |  | + |  | + | blue |
| O95398-3 | Rap guanine nucleotide exchange factor 3 | RAPGEF3 | -0.89 ±0.53 |  | -2.91 ±1.23 | 4.65 | + | + |  | + |  | + | turquoise |
| O15197 | Ephrin type-B receptor 6 | EPHB6 | -1.74 ±0.76 |  | -2.91 ±2.31 | 2.91 | + | + |  | + |  | + | brown |
| P30613-2 | Pyruvate kinase PKLR | PKLR | -0.19 ±0.79 | 0.08 | -2.91 ±0.93 | 5.08 | + | + | + | + |  | + | blue |
| O96013 | Serine/threonine-protein kinase PAK 4 | PAK4 | 0.21 ±0.94 | 0.09 | -2.91 ±1.25 | 4.39 | + | + |  | + |  | + | turquoise |
| E7EX90 | Dynactin subunit 1 | DCTN1 | -0.42 ±0.39 |  | -2.85 ±0.12 | 7.58 | + | + | + | + |  | + | turquoise |
| Q9NQE9 | Histidine triad nucleotide-binding protein 3 | HINT3 | -1.14 ±0.52 | 0.79 | -2.83 ±0.56 | 2.92 | + | + | + | + |  | + | turquoise |
| O94919 | Endonuclease domain-containing 1 protein | ENDOD1 | -2.51 ±1.26 | 2.18 | -2.78 ±0.3 | 2.88 | + | + | + | + |  | + | red |
| F8WC39 | Glycerol kinase | GK | -0.7 ±0.29 |  | -2.75 ±0.71 | 4.6 | + | + |  | + |  | + | blue |
| A0A096LP62 | Inter-alpha-trypsin inhibitor heavy chain H5 | ITIH5 | 0.63 ±0.66 |  | -2.75 ±0.45 | 6.2 | + | + |  | + |  | + | grey |
| E7EVJ5 | Cytoplasmic FMR1-interacting protein 2 | CYFIP2 | -0.15 ±0.84 |  | -2.73 ±0.09 | 3.78 | + | + | + | + |  | + | turquoise |
| Q96BW5-2 | Phosphotriesterase-related protein | PTER | -1.4 ±0.34 | 0.96 | -2.73 ±0.46 | 2.73 | + | + | + | + |  | + | turquoise |
| P15907 | Beta-galactoside alpha-2,6-sialyltransferase 1 | ST6GAL1 | -1.75 ±1.14 |  | -2.71 ±0.1 | 3.27 |  | + | + | + |  | + | brown |
| Q9Y2E5 | Epididymis-specific alpha-mannosidase | MAN2B2 | -1.87 ±0.03 | 0.9 | -2.71 ±0.74 | 2.54 | + | + | + | + |  | + | brown |
| O60637-3 | Tetraspanin-3 | TSPAN3 | -0.58 ±0.2 | 0.43 | -2.68 ±0.62 | 3.38 | + | + | + | + |  | + | greenyellow |
| Q02790 | Peptidyl-prolyl cis-trans isomerase FKBP4 | FKBP4 | 0.28 ±0.16 |  | -2.65 ±0.43 | 3.79 | + | + | + | + |  | + | blue |
| P12955 | Xaa-Pro dipeptidase | PEPD | -0.64 ±0.26 | 0.37 | -2.59 ±0.2 | 2.7 | + | + | + | + |  | + | blue |
| E7EVZ1 | Zinc finger homeobox protein 4 | ZFHX4 | -0.3 ±0.28 |  | -2.58 ±0.86 | 3.63 | + |  |  | + |  | + | turquoise |
| D6R9I9 | ATP-binding cassette sub-family E member 1 | ABCE1 | 0.58 ±0.25 |  | -2.58 ±0.49 | 2.52 | + | + | + | + |  | + | purple |
| E5RHG8 | Transcription elongation factor B polypeptide 1 | TCEB1 | -0.71 ±1.04 | 0.58 | -2.53 ±0.61 | 4.25 | + | + |  | + |  | + | turquoise |
| Q9H993 | Protein-glutamate O-methyltransferase | ARMT1 | -0.13 ±0.08 |  | -2.45 ±0.67 | 2.93 | + | + | + | + |  | + | blue |
| D6RDU5 | Septin-11 | SEPT11 | 0.55 ±0.38 |  | -2.41 ±0.83 | 5.5 | + | + |  | + |  | + | turquoise |
| P31689 | DnaJ homolog subfamily A member 1 | DNAJA1 | -0.04 ±0.24 | 0.01 | -2.39 ±1.1 | 3.03 | + | + |  | + |  | + | turquoise |
| H3BPK3 | Hydroxyacylglutathione hydrolase, mitochondrial | HAGH | -0.61 ±0.47 | 0.48 | -2.39 ±0.2 | 4.32 | + | + | + | + |  | + | turquoise |
| P36507 | Dual specificity mitogen-activated protein kinase kinase 2 | MAP2K2 | -0.78 ±0.42 |  | -2.37 ±0.79 | 3.73 | + | + |  | + |  | + | turquoise |
| O94903 | Proline synthase co-transcribed bacterial homolog protein | PROSC | 0.24 ±0.23 |  | -2.35 ±1.47 | 3.31 |  |  |  | + |  | + | turquoise |
| P49189 | 4-trimethylaminobutyraldehyde dehydrogenase | ALDH9A1 | 0.57 ±0.48 | 0.32 | -2.34 ±1.25 | 2.61 | + | + | + | + |  | + | blue |
| Q15848 | Adiponectin | ADIPOQ | -2.67 ±0.09 | 2.55 | -2.34 ±0.57 | 3.86 | + | + |  | + |  | + | brown |
| Q9UHI8 | A disintegrin and metalloproteinase with thrombospondin motifs 1 | ADAMTS1 | -0.54 ±0.17 |  | -2.32 ±1.54 | 2.74 |  |  |  | + |  | + | red |
| Q58FF8 | Putative heat shock protein HSP 90-beta 2 | HSP90AB2P | 0.42 ±0.1 |  | -2.3 ±0.4 | 2.67 | + | + | + | + |  | + | blue |
| P09619 | Platelet-derived growth factor receptor beta | PDGFRB | -1.72 ±0.9 |  | -2.29 ±0.76 | 4.19 |  | + |  | + |  | + | tan |
| Q9BZV1 | UBX domain-containing protein 6 | UBXN6 | 0.33 ±0.26 |  | -2.29 ±0.5 | 3.09 | + | + | + | + |  | + | turquoise |
| P40189-3 | Interleukin-6 receptor subunit beta | IL6ST | -2.2 ±1.48 | 1.83 | -2.28 ±1.76 | 2.76 | + | + |  | + |  | + | brown |
| E9PP76 | Superoxide dismutase [Cu-Zn] | CCS | -0.3 ±0.02 |  | -2.25 ±0.46 | 5.04 |  |  |  | + |  | + | grey |
| P17252 | Protein kinase C alpha type | PRKCA | -0.56 ±0.26 |  | -2.2 ±0.93 | 3.03 | + | + |  | + |  | + | turquoise |
| P21709-3 | Ephrin type-A receptor 1 | EPHA1 | -2.7 ±0.86 |  | -2.17 ±1.3 | 2.42 |  | + |  | + |  | + | brown |
| Q14376 | UDP-glucose 4-epimerase | GALE | -1.04 ±0.2 | 0.61 | -2.15 ±0.25 | 2.54 | + | + | + | + |  | + | turquoise |
| G3V3U4 | Proteasome subunit alpha type | PSMA6 | 1.28 ±0.09 | 0.95 | -2.14 ±0.26 | 3 | + | + | + | + |  | + | blue |
| Q9BTY2 | Plasma alpha-L-fucosidase | FUCA2 | -1.65 ±0.41 | 0.97 | -2.14 ±0.35 | 2.41 | + | + | + | + |  | + | brown |
| O15031 | Plexin-B2 | PLXNB2 | -1.18 ±0.34 | 0.93 | -2.08 ±1.6 | 2.89 | + | + | + | + |  | + | turquoise |
| P49588 | Alanine--tRNA ligase, cytoplasmic | AARS | -1.4 ±0.39 | 1.62 | -2.06 ±0.57 | 2.53 | + | + | + | + |  | + | blue |
| D6RB59 | Exocyst complex component 3 | EXOC3 | 0.48 ±0.55 |  | -2.06 ±0.76 | 3.48 | + | + |  | + |  | + | turquoise |
| P00167-2 | Cytochrome b5 | CYB5A | -0.18 ±0.04 |  | -2.03 ±0.53 | 3.63 |  |  |  | + |  | + | turquoise |
| E9PIR7 | Thioredoxin reductase 1, cytoplasmic | TXNRD1 | -0.55 ±0.13 |  | -2.02 ±0.55 | 2.74 | + | + | + | + |  | + | blue |
| Q92887 | Canalicular multispecific organic anion transporter 1 | ABCC2 | -0.87 ±0.19 |  | -2 ±0.24 | 5.5 |  | + |  | + |  | + | brown |
| A0A087X0R6 | Sorting nexin-12 | SNX12 | 0.75 ±0.03 |  | -1.99 ±0.01 | 3.26 | + | + | + | + |  | + | turquoise |
| Q9HAT8 | E3 ubiquitin-protein ligase pellino homolog 2 | PELI2 | 0.35 ±0.46 |  | -1.99 ±0.88 | 3.02 |  |  |  | + |  | + | grey |
| A0A087WVM2 | CD177 antigen | CD177 | -1.05 ±0.86 |  | -1.9 ±0.66 | 2.53 | + | + |  | + |  | + | black |
| P28074 | Proteasome subunit beta type-5 | PSMB5 | 0.44 ±0.09 |  | -1.86 ±0.89 | 3.06 | + | + |  | + |  | + | blue |
| A0A0A0MSE9 | Pleckstrin homology domain-containing family B member 2 | PLEKHB2 | -1.14 ±0.04 | 0.59 | -1.8 ±0.35 | 4.24 | + | + |  | + |  | + | turquoise |
| P28072 | Proteasome subunit beta type-6 | PSMB6 | 1.59 ±0.74 | 1.46 | -1.71 ±0.47 | 3.65 | + | + |  | + |  | + | grey |
| P82980 | Retinol-binding protein 5 | RBP5 | -1.65 ±0.88 | 1.38 | -1.52 ±0.42 | 2.58 | + | + | + | + |  | + | black |
| P11908 | Ribose-phosphate pyrophosphokinase 2 | PRPS2 | -1.35 ±0.42 |  | -1.47 ±0.43 | 3.47 | + | + |  | + |  | + | turquoise |
| F8W810 | Putative eukaryotic translation initiation factor 2 subunit 3-like protein | EIF2S3L | -0.27 ±0.22 |  | -1.42 ±0.63 | 2.63 | + | + |  | + |  | + | blue |
| P0C0L4 | Complement C4-A | C4A | 0.66 ±0.11 | 0.51 | 1.42 ±0.04 | 2.55 | + | + | + | + |  | + | green |
| E7END6 | Vitamin K-dependent protein C | PROC | 0.81 ±1.1 |  | 2.08 ±0.39 | 4.04 | + |  | + |  |  | + | grey |
| A0A1W2PQB1 | Low affinity immunoglobulin gamma Fc region receptor III-A | FCGR3A | 1.05 ±1.23 |  | 2.17 ±0.48 | 2.65 | + |  | + | + |  | + | grey |
| A6QRJ0 | Deoxyribonuclease-1-like 1 | DNASE1L1 | -1.62 ±0.74 |  | 2.43 ±0.13 | 9.19 |  | + | + |  |  | + | grey |
| P17213 | Bactericidal permeability-increasing protein | BPI | 1.35 ±1.36 |  | 2.48 ±0.69 | 2.6 | + | + | + | + |  | + | green |
| P02743 | Serum amyloid P-component | APCS | 1.11 ±0.29 | 1.01 | 2.68 ±0.01 | 3.54 | + | + | + | + |  | + | green |
| B1AMW1 | Lymphocyte function-associated antigen 3 | CD58 | 0.05 ±0.55 |  | 2.85 ±0.59 | 3.52 | + | + | + | + |  | + | grey |
| Q96KN2 | Beta-Ala-His dipeptidase | CNDP1 | 0.05 ±0.43 | 0.02 | 2.97 ±1.59 | 4.12 | + | + | + | + |  | + | green |
| P02679 | Fibrinogen gamma chain | FGG | 2.35 ±0.53 | 1.63 | 2.98 ±0.45 | 3.01 | + | + | + | + |  | + | green |
| P02675 | Fibrinogen beta chain | FGB | 2.96 ±0.12 | 2.19 | 3.02 ±0.11 | 2.78 | + | + | + | + |  | + | green |
| P07360 | Complement component C8 gamma chain | C8G | 1.2 ±0.61 |  | 3.03 ±0.73 | 5.52 | + | + | + |  |  | + | green |
| P00739 | Haptoglobin-related protein | HPR | 2.58 ±1.51 | 1.56 | 3.1 ±1.81 | 2.83 | + | + | + | + |  | + | green |
| V9GYM3 | Apolipoprotein A-II | APOA2 | 1.66 ±0.61 | 1.6 | 3.23 ±1.32 | 3.89 | + | + | + | + |  | + | magenta |
| P03951-2 | Coagulation factor XI | F11 | -0.18 ±0.63 | 0.11 | 3.27 ±0.78 | 5.4 | + | + | + |  |  | + | greenyellow |
| P27701-2 | CD82 antigen | CD82 | -0.39 ±0.65 | 0.24 | 3.33 ±1.15 | 5.99 | + | + | + | + |  | + | pink |
| Q9Z2K1 |  | KRT16 | 0.55 ±0.09 |  | 3.35 ±0.95 | 6.26 | + | + | + | + |  | + | grey |
| P01624 | Ig kappa chain V-III region POM | IGKV3OR2-268 | -1.81 ±0.19 | 0.81 | 3.41 ±3.08 | 2.54 | + | + | + | + |  | + | magenta |
| Q8ND23-3 | Capping protein, Arp2/3 and myosin-I linker protein 3 | CARMIL3 | -0.02 ±1.16 |  | 4.08 ±1.14 | 7.07 | + |  | + |  |  | + | grey |
| C9JP03 | SLAIN motif-containing protein 1 | SLAIN1 | -0.88 ±0.17 | 0.26 | 5.18 ±2.04 | 6.08 | + | + | + |  |  | + | purple |
| P04114 | Apolipoprotein B-100 | APOB | 3.26 ±0.24 | 1.41 | 5.49 ±0.49 | 3.04 | + | + | + | + |  | + | green |
| I3L3J2 | Zinc finger and SCAN domain-containing protein 32 | ZSCAN32 | -0.03 ±0.35 |  | 7.31 ±0.95 | 14.39 |  |  | + |  |  | + | grey |
| Q9H9A6 | Leucine-rich repeat-containing protein 40 | LRRC40 | -4.54 ±2.04 | 4.56 | -4.36 ±1.38 | 6.58 |  | + |  | + | + | + | grey |
| Q8N122-3 | Regulatory-associated protein of mTOR | RPTOR | -4.38 ±2.24 | 4.13 | -2.91 ±1.76 | 3.17 |  | + | + | + | + | + | grey |
| C9JD53 | Isopentenyl-diphosphate Delta-isomerase 1 | IDI1 | -2.76 ±0.75 | 3.12 | -3.18 ±1.62 | 4.43 | + | + |  | + | + | + | brown |
| Q02487-2 | Desmocollin-2 | DSC2 | -2.69 ±0.72 | 2.81 | -4.04 ±2.02 | 4.77 | + | + |  | + | + | + | brown |
| P07225 | Vitamin K-dependent protein S | PROS1 | 3.42 ±1.45 | 3.94 | 3.19 ±1.28 | 4.43 | + | + | + |  | + | + | green |
| P04003 | C4b-binding protein alpha chain | C4BPA | 3.72 ±0.3 | 4.28 | 3.09 ±2.31 | 3.57 | + | + | + |  | + | + | green |
| Q5S007 | Leucine-rich repeat serine/threonine-protein kinase 2 | LRRK2 | -7.09 ±0.51 | 4.63 | -3.28 ±1 |  | + | + | + | + | + |  | turquoise |
| Q6NXT6-2 | Transmembrane anterior posterior transformation protein 1 homolog | TAPT1 | -5.39 ±2.66 | 4.91 | 0.68 ±0.15 | 0.27 |  | + | + | + | + |  | red |
| Q9UJU2-4 | Lymphoid enhancer-binding factor 1 | LEF1 | -4.62 ±2.02 | 4.79 | -1.48 ±1.35 | 1.3 |  | + | + | + | + |  | red |
| A4FU69-2 | EF-hand calcium-binding domain-containing protein 5 | EFCAB5 | -4.14 ±1.07 | 3.23 | 1.19 ±0.28 |  | + | + | + | + | + |  | magenta |
| A0A0C4DG40 | Nesprin-1 | SYNE1 | -4.13 ±1.8 | 5.57 | 0.72 ±0.61 |  | + | + | + | + | + |  | grey |
| Q8NDA2-2 | Hemicentin-2 | HMCN2 | -3.78 ±2.62 | 3.83 | 0.25 ±0.01 |  |  | + |  |  | + |  | salmon |
| P55291 | Cadherin-15 | CDH15 | -3.66 ±0.59 | 4.88 | -0.89 ±1.97 | 0.43 | + | + | + | + | + |  | brown |
| P11678 | Eosinophil peroxidase | EPX | -3.62 ±0.18 | 9.52 | 0.39 ±0.1 |  | + | + | + | + | + |  | pink |
| A0A075B7D9 | RNA-binding protein FUS | TAF15 | -3.58 ±1.6 | 4.09 | -0.8 ±0.44 | 0.23 |  | + | + | + | + |  | red |
| O00182-3 | Galectin-9 | LGALS9 | -3.48 ±0.32 | 3.61 | -1.17 ±0.22 | 0.77 | + | + | + | + | + |  | brown |
| Q13733 | Sodium/potassium-transporting ATPase subunit alpha-4 | ATP1A4 | -3.46 ±0.66 | 5.56 | -0.33 ±0.2 |  | + | + | + | + | + |  | pink |
| Q08554-2 | Desmocollin-1 | DSC1 | -3.41 ±1.01 | 3.27 | -1.52 ±1.75 |  | + | + |  | + | + |  | purple |
| Q9UKM7 | Endoplasmic reticulum mannosyl-oligosaccharide 1,2-alpha-mannosidase | MAN1B1 | -3.19 ±1.01 | 3.49 | 0.36 ±0.19 |  | + | + |  |  | + |  | pink |
| A0A024R412 | Neuropilin-2 | NRP2 | -3.17 ±0.67 | 6.58 | 0.27 ±0.08 |  |  | + |  |  | + |  | purple |
| Q9NSK0 | Kinesin light chain 4 | KLC4 | -3.17 ±1.12 | 4.86 | -1.1 ±0.23 | 0.75 |  | + | + | + | + |  | grey |
| Q9NQX4 | Unconventional myosin-Vc | MYO5C | -3.08 ±1.17 | 3.38 | 1.27 ±0.93 | 0.62 | + | + | + | + | + |  | turquoise |
| O00187-2 | Mannan-binding lectin serine protease 2 | MASP2 | -2.95 ±0.15 | 5.45 | -1.44 ±0.31 | 2.38 | + | + | + | + | + |  | brown |
| P08572 | Collagen alpha-2(IV) chain | COL4A2 | -2.93 ±0.03 | 2.96 | -1.22 ±0.79 | 0.61 | + | + | + | + | + |  | red |
| Q6UWV6 | Ectonucleotide pyrophosphatase/phosphodiesterase family member 7 | ENPP7 | -2.73 ±0.29 | 2.96 | 0 ±0.65 | 0 | + | + | + | + | + |  | grey |
| X6R868 | Carboxylic ester hydrolase | CEL | -2.64 ±1.01 | 3.41 | -0.03 ±0.39 | 0.01 | + | + | + | + | + |  | brown |
| P41181 | Aquaporin-2 | AQP2 | -2.63 ±0.69 | 2.91 | -0.44 ±0.62 | 0.27 | + | + | + | + | + |  | turquoise |
| P20160 | Azurocidin | AZU1 | -2.3 ±0.59 | 3.73 | 0.16 ±0.28 | 0.06 |  | + | + | + | + |  | grey |
| Q16769 | Glutaminyl-peptide cyclotransferase | QPCT | -2.26 ±0.71 | 2.96 | -0.75 ±0.91 | 0.55 | + | + | + | + | + |  | tan |
| P51151 | Ras-related protein Rab-9A | RAB9A | -2.12 ±0.11 | 4.01 | -0.46 ±0.46 | 0.37 |  | + | + | + | + |  | turquoise |
| Q9UKU9 | Angiopoietin-related protein 2 | ANGPTL2 | -2.04 ±0.2 | 2.86 | -1.26 ±0.74 | 0.97 | + | + | + | + | + |  | brown |
| P31371 | Fibroblast growth factor 9 | FGF9 | -2.03 ±1.3 | 2.94 | 1.53 ±1.81 |  |  | + | + |  | + |  | purple |
| P22894 | Neutrophil collagenase | MMP8 | 2.28 ±1.36 | 3.18 | 0.85 ±0.61 |  | + |  | + |  | + |  | green |
| P15104 | Glutamine synthetase | GLUL | 2.93 ±1.58 | 4.2 | 1.17 ±0.91 |  | + |  | + |  | + |  | yellow |
| P20851-2 | C4b-binding protein beta chain | C4BPB | 2.93 ±0.74 | 5.72 | 1.53 ±1.49 |  | + |  | + |  | + |  | green |
| O00602 | Ficolin-1 | FCN1 | 3.03 ±1.03 | 5.97 | 1.08 ±1.28 |  | + |  | + |  | + |  | green |
| A8MTJ3 | Guanine nucleotide-binding protein G(t) subunit alpha-3 | GNAT3 | 3.04 ±1.58 | 3.3 | 0.28 ±0.02 |  | + | + | + | + | + |  | yellow |
| A0A0A0MRJ7 | Coagulation factor V | F5 | 3.19 ±2.13 | 3.51 | 1.11 ±0.88 |  | + |  | + |  | + |  | green |
| J3KNB4 | Cathelicidin antimicrobial peptide | CAMP | 3.28 ±1.25 | 2.84 | 2.52 ±1.47 | 1.93 | + | + | + | + | + |  | green |
| A0A087WUZ3 | Spectrin beta chain, non-erythrocytic 1 | SPTBN1 | 3.5 ±1.69 | 4.56 | -0.51 ±0.11 |  | + |  | + | + | + |  | blue |
| P07237 | Protein disulfide-isomerase | P4HB | 3.53 ±1.44 | 4.7 | 0.48 ±0.34 | 0.26 | + |  | + | + | + |  | grey |
| P28070 | Proteasome subunit beta type-4 | PSMB4 | 3.63 ±0.66 | 7.03 | 1.85 ±1.34 |  | + |  | + |  | + |  | green |
| O14791 | Apolipoprotein L1 | APOL1 | 3.68 ±2.74 | 3.05 | 1.91 ±1.38 |  | + |  | + | + | + |  | green |
