# Supplementary material for: Proteomic Analysis of Urinary Extracellular Vesicles Reveals a Role for the Complement System in Medullary Sponge Kidney Disease
Source: Int J Mol Sci. 2019 Nov 5;20(21):5517. doi: 10.3390/ijms20215517 (PMC6862015; doi:10.3390/ijms20215517)
Supplement: Supplementary file 1 [file ijms-20-05517-s001.zip › Supplementary material_Bruschi et al_REVISED.pdf]

Supplementary material

Proteomic analysis of urinary extracellular vesicles reveals a role for the complement system in Medullary Sponge Kidney disease.

Maurizio Bruschi[1*], Simona Granata[2*], Giovanni Candiano[1], Antonia Fabris[2], Andrea Petretto[3], Gian Marco Ghiggeri[4], Giovanni Gambaro[2], Gianluigi Zaza[2]

[1] Laboratory of Molecular Nephrology, IRCCS Istituto Giannina Gaslini, Genoa, Italy

[2] Renal Unit, Department of Medicine, University Hospital of Verona, Italy

[3] Laboratory of Mass Spectrometry - Core Facilities, IRCCS Istituto Giannina Gaslini, Genova, Italy

[4] Division of Nephrology, Dialysis and Transplantation, IRCCS Istituto Giannina Gaslini, Genoa, Italy

Supplementary Figures

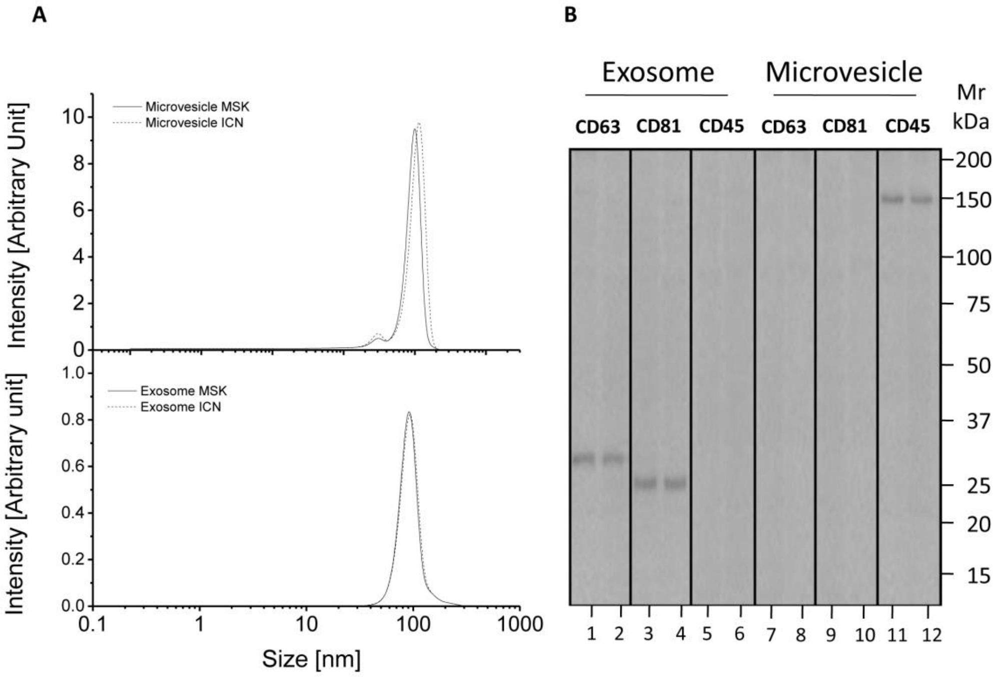

<u>Figure S1</u>. Characterization of exosome and microvesicle fractions purified from the urine samples of ICN and MSK patients. (A) Plot showing the size distribution of microvesicles (top panel) and exosomes (bottom panel) as evaluated by dynamic light scattering. The plot shows a Gaussian distribution profile with mean peaks at 90 ± 5 and 1000 ± 65 nm for exosomes or microvesicles, respectively. No statistical differences were observed between the exosomes or microvesicles isolated from ICN and MSK patients. **(B)** Representative western blot analysis showing the antigen profiles of exosomes and microvesicles isolated from the urine of ICN and MSK patients. Whole exosomes (lanes 1–6) and microvesicles (lanes 7–12) from ICN (lanes 1, 3, 5, 7, 9 and 11) and MSK (lanes 2, 4, 6, 8, 10 and 12) patients were analyzed by detecting CD63 (lanes 1–2, 7–8), CD81 (lanes 3–4, 9–10) and CD45 (lanes 5–6, 11–12). Stain-free technology was used as a loading control. No difference in size or antigen profile was observed between ICN and MSK patients for either type of vesicle. Western blot analysis also revealed that the exosomes were positive for CD63 and CD81 but not CD45, whereas the microvesicles showed the opposite antigen profile.

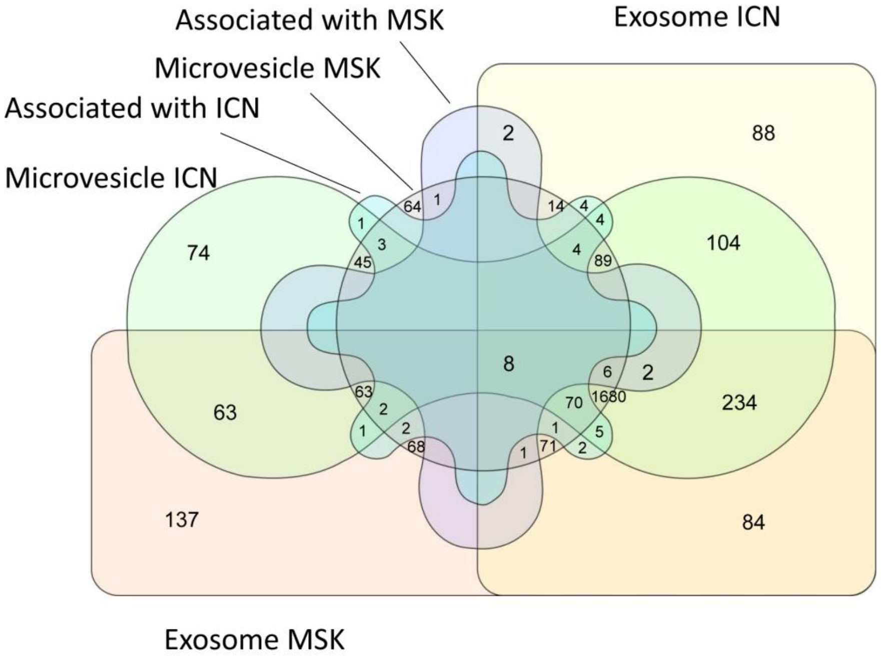

__Figure S2__. Venn diagram of all proteins identified in exosomes and microvesicles isolated from the urine of ICN and MSK patients, and proteins associated with ICN and MSK. Venn diagram shows common and exclusive proteins identified in our study and the proteins already described for ICN and MSK. The numbers represent the distinct proteins in the overlapping and non-overlapping areas. A total of 119 proteins have already been associated with ICN or MSK (DisGeNET database).

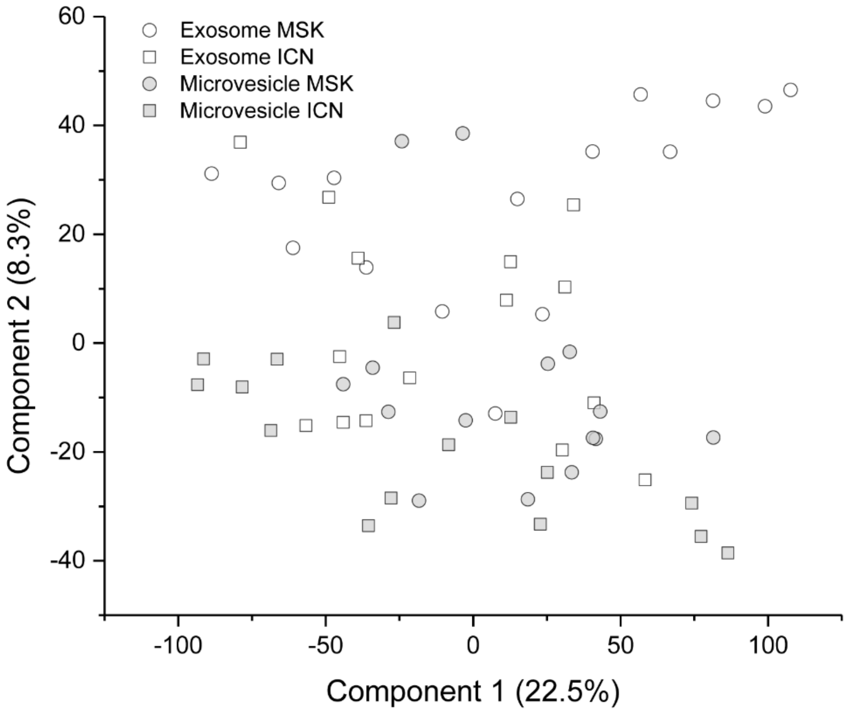

<u>Figure S3.</u> Multidimensional scaling analysis of extracellular vesicles from the urine of ICN and MSK patients. The plot shows the unsupervised clustering of exosomes (white symbols) and microvesicles (gray symbols) from MSK (circles) and ICN (squares) patients using all identified proteins. No outliers were detected.

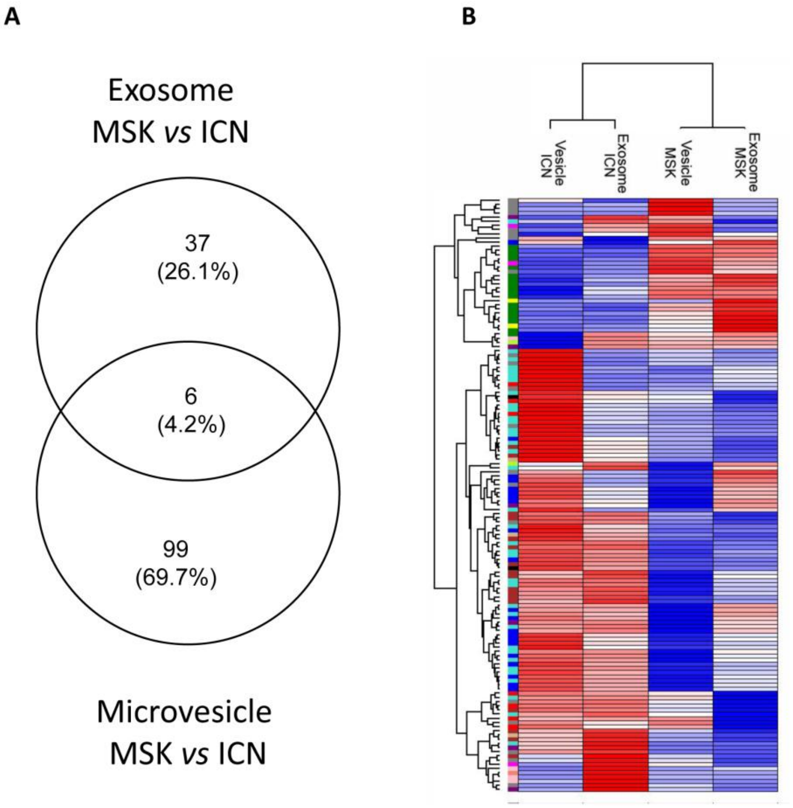

<u>Figure S4</u>. Exosome and microvesicle proteins that show significant differences in abundance between ICN and MSK patients. (A) Venn diagram of common and exclusive exosome and microvesicle proteins from the urine of ICN and MSK patients, showing the numbers (and percentages) of distinct proteins in the overlapping and non-overlapping areas. **(B)** Heat map of 142 proteins identified through the use of univariate statistical analysis. In the heat map, each row represents a protein, and each column represents a sample type. Normalized Z-scores of protein abundance are depicted by a pseudocolor scale with red indicating positive expression, white equal expression, and blue negative expression compared to each protein value, whereas the dendrogram displays the outcome of unsupervised hierarchical clustering, placing similar proteome profile values near each other. Visual inspection of the dendrogram and heat map confirms the ability of these proteins to distinguish between the four sample types.

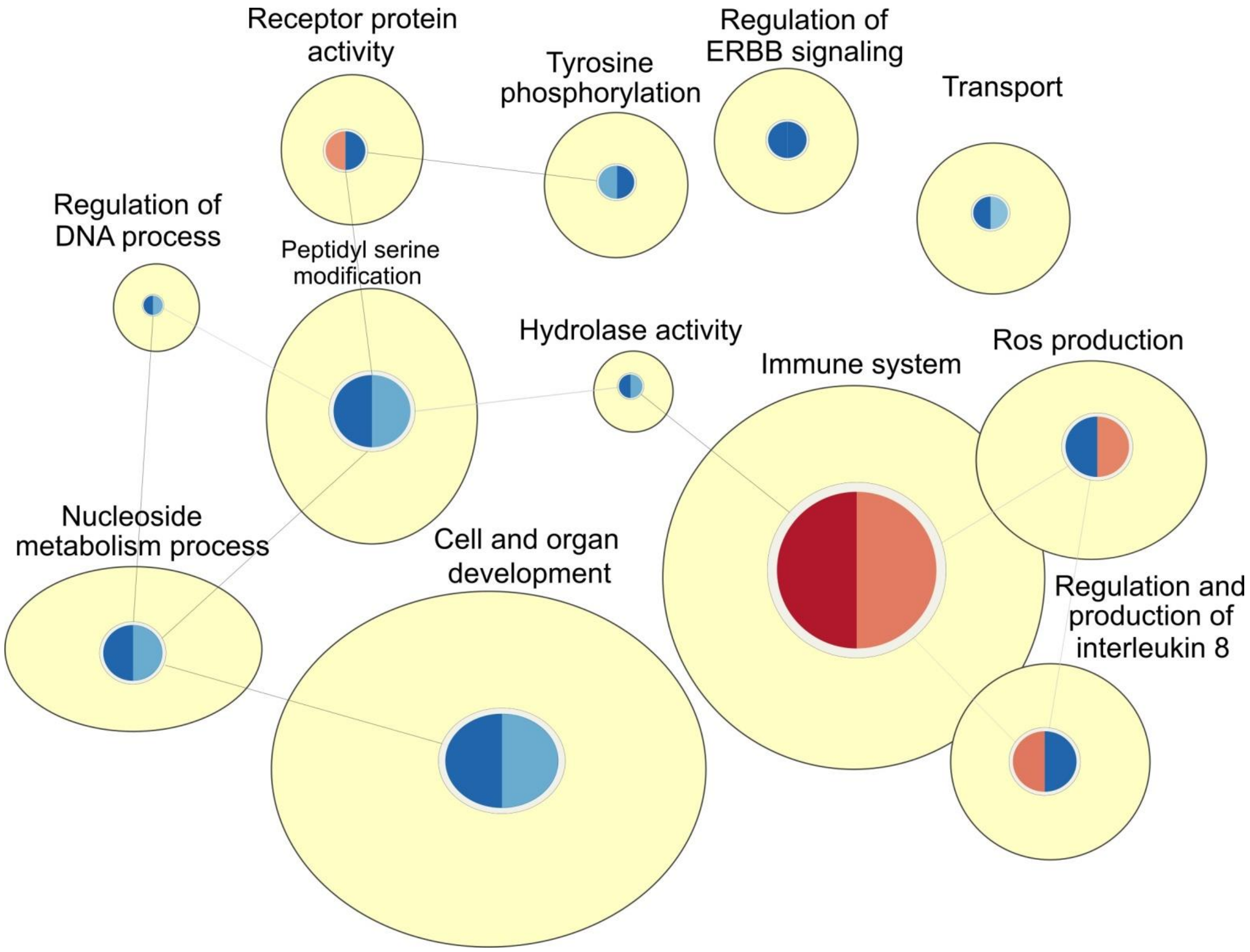

<u>Figure S5</u>. Enrichment network map of the significant proteins found in urinary microvesicles and exosomes from MSK and ICN patients. Nodes (circles) and edges (lines) represent the biological processes and their interactions, respectively. Node color represents the enrichment score in the urinary exosome (darker) or microvesicle (lighter) of MSK (red) or ICN (blue) patients, whereas node size represents their significance (FDR q-value). The pathways are grouped into 12 clusters (ellipses) based on Gene Ontology functional annotations.
